# Tau protein as a regulator of mitochondrial function and dynamics

**DOI:** 10.1073/pnas.2521642123

**Published:** 2026-06-30

**Authors:** Eleni Tsakiri, Carlos Campos-Marques, Christina Ploumi, Kalliopi Skourti, Antonis Roussos, Eirini Mytilinaiou, Anastasia Vamvaka Iakovou, Ildete Luísa Ferreira, Chrysoula Dioli, Despoina D. Gianniou, Martina Samiotaki, Jonas Campos, Clarissa Waites, Nuno Sousa, Ioannis P. Trougakos, Joana M. Silva, A. Cristina Rego, Ioannis Sotiropoulos, Konstantinos Palikaras

**Affiliations:** ^a^ Department of Physiology, Medical School, National and Kapodistrian University of Athens, Athens 11527, Greece; ^b^ Life and Health Sciences Research Institute (ICVS), University of Minho, Braga 4710-057, Portugal; ^c^ICVS/3B’s–PT Government Associate Laboratory, Braga/Guimarães 4710-057, Portugal; ^d^Laboratory of Brain exosomes and Pathology - ExoBrain, Institute of Biosciences and Applications, National Centre for Scientific Research Demokritos, Agia Paraskevi 15341, Greece; ^e^ Center for Neuroscience and Cell Biology, University of Coimbra, Coimbra 3004-504, Portugal; ^f^ Center for Innovative Biomedicine and Biotechnology, University of Coimbra, Coimbra 3004-504, Portugal; ^g^ Institute of Interdisciplinary Research, University of Coimbra, Coimbra 3030-789, Portugal; ^h^ Department of Biology, National and Kapodistrian University of Athens, Athens 15784, Greece; ^i^Biomedical Sciences Research Center “Alexander Fleming”, Institute for Bio-innovation, Vari 16672, Greece; ^j^ Department of Pathology and Cell Biology, Taub Institute for Research on Alzheimer’s Disease and Aging Brain, Columbia University Irving Medical Center, New York, NY 10032; ^k^ Department of Neuroscience, Columbia University, New York, NY 10032; ^l^Centro Universitário de Jaguariúna, São Paulo 13918-112, Brazil; ^m^Centro Universitário Max-Planck, São Paulo 13343-060, Brazil; ^n^ Faculty of Medicine, University of Coimbra, Coimbra 3000-354, Portugal

**Keywords:** mitochondria, mitochondrial dynamics, Tau, neuron, neurodegeneration

## Abstract

Mitochondrial damage is a shared hallmark of brain aging and neurodegeneration. While pathological Tau mutations disrupt mitochondrial dynamics and function, the physiological role of wild-type (WT) Tau in the maintenance of mitochondrial homeostasis remains poorly understood. Here, using *Caenorhabditis elegans* and mice lacking PTL-1, the nematode Tau-like homolog, and Tau respectively, we demonstrate that Tau deficiency promotes a shift toward a pro-fusion mitochondrial state associated with enhanced mitochondrial function and stress resistance. In both models, loss of Tau leads to increased mitochondrial activity and altered redox homeostasis, while it enhances resistance to heat and mitochondrial stress in *C. elegans*. Strikingly, loss of FZO-1, the mitofusin homolog, abolishes the beneficial phenotypes, whereas its overexpression phenocopies key aspects of Tau/PTL-1 deficiency. Together, our findings uncover a conserved role for WT Tau in restraining mitochondrial fusion and functional adaptation, highlighting its contribution to mitochondrial homeostasis and cellular stress responses.

## Significance

Mitochondrial dynamics are essential for neuronal health, enabling cells to adapt energy production and stress responses to changing demands. Although pathological Tau has been extensively linked to mitochondrial dysfunction promoting neurodegeneration, its physiological role remains elusive. Here, using complementary studies in mice and *Caenorhabditis elegans*, we demonstrate that loss of Tau or its homolog PTL-1 promotes mitochondrial fusion and enhances stress resilience through a conserved mitofusin-dependent mechanism. These findings reveal an unexpected role for wild-type Tau as a regulator of mitochondrial dynamics and functional adaptation, providing insight into how Tau contributes to neuronal homeostasis beyond its pathological aggregation.

Beyond its primary role in regulating microtubule dynamics and cytoskeletal stability (1–3), Tau protein has recently been involved in additional neuronal functions, including the maintenance of genomic integrity (4, 5) and the regulation of lipid droplet biogenesis, a process that mitigates oxidative stress and neuronal toxicity caused by reactive oxygen species (ROS), thereby safeguarding brain function (6). Previous studies have linked Tau dysfunction to impaired intraneuronal trafficking and/or mitochondrial malfunction in Alzheimer’s disease (AD), neurodegeneration (7–10), and stress-induced brain pathology (11–17), highlighting a critical interplay between Tau and mitochondrial dynamics. Interestingly, Tau has been shown to interact with mitochondrial proteins and localize within specific mitochondrial subcompartments (18, 19). Notably, Tau exhibits stronger interactions with mitochondrial proteins (e.g., cytochrome C and BCS1L) in wild-type (WT) human neurons compared to neurons harboring pathogenic Tau mutations (19). Moreover, overexpression of the mutant P301L-Tau in mouse impaired mitochondrial function by disrupting inner membrane components, particularly complexes I and V (20). Consistently, proteomic analyses in P301L-Tau-expressing mice and cell models revealed reduced protein levels of respiratory complexes (20, 21). Similarly, Tau oligomers were shown to disrupt mitochondrial function and were associated with learning deficits in mice (22).

Due to their high energy demands, neurons are particularly vulnerable to mitochondrial defects. Impaired mitochondria result in reduced ATP production, loss of membrane potential, and elevated ROS generation, all of which compromise neuronal survival (23–25). To counteract these effects, cells activate mitochondrial quality control mechanisms, including proteasomal degradation, mitochondrial proteases, mitochondrial-derived vesicles, fission-fusion dynamics, and mitophagy (26). Mitochondrial fission, often coupled with mitophagy, facilitates the clearance of damaged mitochondria by segmenting them into smaller units that are more easily degraded (27). Emerging evidence indicates that Tau dysfunction disrupts the quality control processes by altering mitochondrial dynamics and clearance. For instance, overexpression of WT Tau or the pathogenic P301L-Tau variant inhibits mitophagy in *Caenorhabditis elegans* and mammalian cells by interfering with Parkin translocation to defective organelles (28–30). Additionally, hyperphosphorylated Tau interacts with Dynamin-related protein 1 (Drp1), impairing mitochondrial fission and contributing to mitochondrial abnormalities observed in AD models (30). These findings underline the deleterious effects of pathological Tau on mitochondrial integrity and quality control. However, the physiological role of normal, nonpathological Tau in regulating mitochondrial organization, remodeling, and function remains poorly understood. Using both mouse model and the nematode *C. elegans*, we demonstrate that loss of normal Tau protein triggers a pro-fusion mitochondrial state, enhances mitochondrial function and mitophagy, accompanied by altered mitochondrial stress response. These changes are associated with reduced vulnerability to various stressors and occur in an FZO-1/Mitofusins-dependent manner.

## Materials and Methods

Detailed methods are provided in *SI Appendix*, including *C. elegans* strains and transgenic strain generation, mouse lines and husbandry, locomotion and lifespan assays, mitophagy and mitochondrial morphology analyses, RT-qPCR, mitochondrial membrane potential and ROS measurements, heat and mitochondrial stress assays, mitochondrial isolation procedures, Seahorse-based bioenergetic analyses, transmission electron microscopy (TEM), primary neuronal cultures, western blotting, quantitative proteomics, and data processing pipelines. *C. elegans* were maintained using standard procedures on OP50-seeded NGM plates, and all strains used in this study are listed in *SI Appendix*, Table S1. Tau-KO mice and WT littermates were analyzed under approved experimental protocols, as described in *SI Appendix*. Imaging, biochemical, ultrastructural, and proteomic analyses were performed using established methods detailed in *SI Appendix*. Statistical analyses were performed using GraphPad Prism; tests used for each dataset, sample sizes, and replicate information are indicated in the figure legends and *SI Appendix*.

## Results

### Tau Deficiency Enhances Mitochondrial Activity and Sustains Mitophagy.

To investigate the role of Tau in mitochondrial dynamics and function, we utilized two complementary animal models: i) mice lacking the *Mapt* gene (Tau knock-out mice; Tau-KO) (31) and ii) the nematode *C. elegans* carrying a deletion in the *ptl-1* gene [*ptl-1*(*ok621*)], the worm ortholog of Tau (hereafter referred to as Ptl1-KO). Notably, the *C. elegans* PTL-1 protein shares a high degree of sequence homology with the mammalian Tau within the microtubule-binding domains (*SI Appendix,* Fig. S1*A*).

**Fig. 1.**
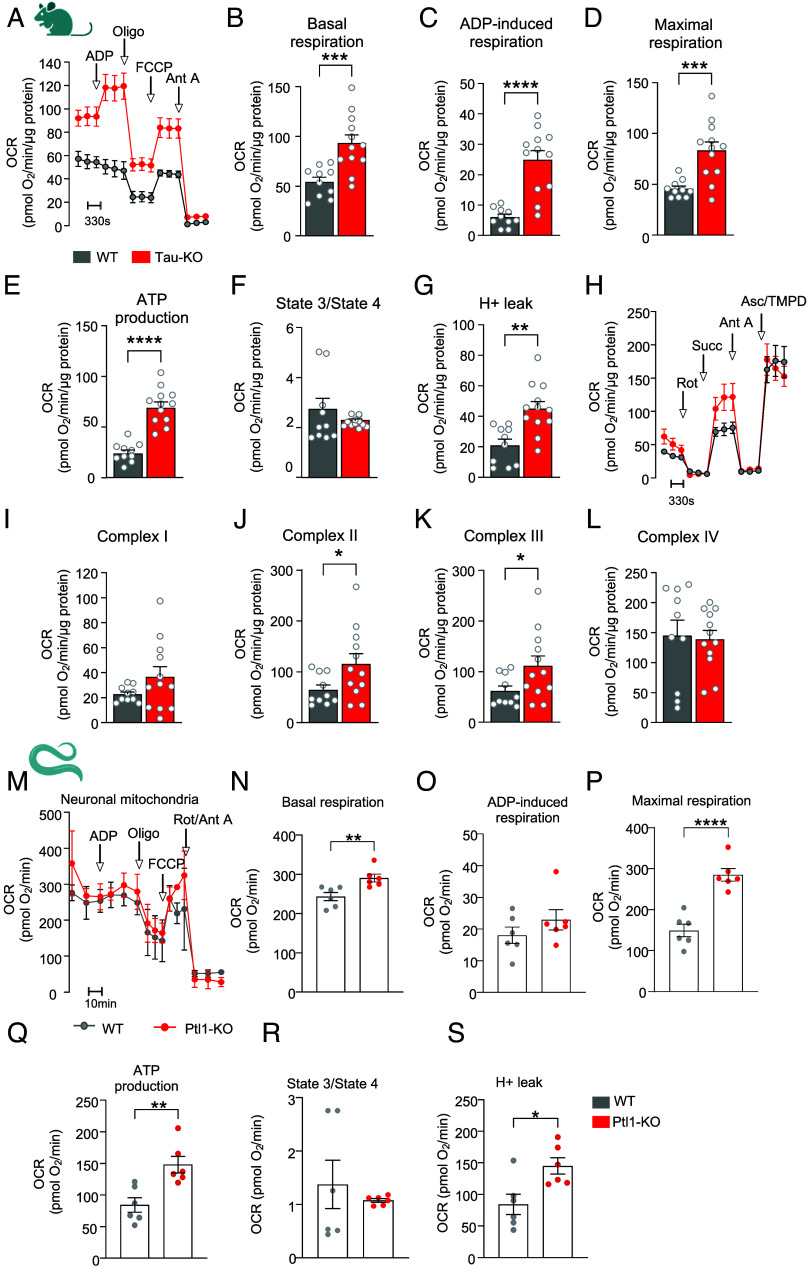
Loss of Tau/PTL-1 is associated with increased mitochondrial respiratory activity in the mouse brain and *C. elegans* neurons. (*A–L*) Brain mitochondria isolated from WT and Tau-KO mice were analyzed by Seahorse XF to monitor mitochondrial bioenergetics in real time. OCR (pmol/min/µg protein) was measured under sequential treatment with mitochondrial substrates, modulators, and inhibitors (*SI Appendix*, *Materials and Methods* and Fig. S1*B*). Representative OCR traces are shown in *A* and *H*. Under coupled respiration conditions (*A–G*), Tau-KO mitochondria displayed higher OCR under basal (*B*) and ADP-induced respiration (*C*), together with increased ATP-linked respiration (*E*) and H^+^ leak (*G*), while the respiratory control ratio (state 3/state 4) remained unchanged (*F*). Because basal respiration was already elevated in Tau-KO mitochondria, the higher OCR observed after FCCP reflects increased overall respiratory activity under these conditions rather than an isolated increase in maximal respiratory capacity (*D*). Under uncoupled electron flow conditions (*H–L*), complex II- and III-linked respiration were increased in Tau-KO mitochondria (*J* and *K*), whereas complex I- and complex IV-linked respiration were unchanged (*I* and *L*). (*M–S*) Neuronal mitochondria isolated from WT and Ptl1-KO *C. elegans* were analyzed by Seahorse XF for real-time monitoring of mitochondrial bioenergetics. Representative OCR traces are shown in *M*. Ptl1-KO neuronal mitochondria exhibited increased basal OCR (*N*), higher FCCP-stimulated respiration (*P*), as well as increased ATP-linked respiration (*Q*) and H^+^ leak (*S*), whereas ADP-stimulated respiration (*O*) and the respiratory control ratio (state 3/state 4) (*R*) were unchanged. Together, these data indicate that loss of Tau/PTL-1 is associated with enhanced mitochondrial respiratory activity in both mouse and nematode neurons. Data are presented as mean ± SEM, analyzed by the unpaired *t* test (*P < 0.05, **P < 0.01, ***P < 0.001 and ****P < 0.0001). For the respirometric analysis of mouse neuronal mitochondria two independent biological replicates were used (n = 10 to 12 mice/ group), while for the nematodes’ neuronal mitochondria three independent experiments were used (n = 3; N = 50,000 nematodes/group), each analyzed twice (two technical replicates).

To determine how Tau deficiency affects mitochondrial bioenergetics, we performed high-resolution respirometry (Seahorse XF) on mitochondria isolated from the prefrontal cortex of Tau-KO and WT littermate mice. Tau-KO mitochondria exhibited increased respiratory activity, as reflected by higher basal and ADP-stimulated oxygen consumption rates (OCR) (Fig. 1 *A*–*D* and *SI Appendix*, Fig. S1*B*), accompanied by elevated ATP-linked respiration (Fig. 1*E*) and increased proton (H^+^) leak (Fig. 1*G*), while the respiratory control ratio (state 3/state 4) remained unchanged (Fig. 1*F*). Although FCCP treatment resulted in increased OCR in Tau-KO mitochondria (Fig. 1*A*), this effect reflects an overall elevation in respiratory activity rather than a selective increase in maximal respiratory capacity, as basal respiration was already elevated. Under uncoupled electron flow conditions (Fig. 1*H*), complex II- and III- linked respiration were increased in Tau-KO mitochondria (Fig. 1 *J* and *K*), whereas complex I and IV activities remained unchanged (Fig. 1 *I* and *L*).

To assess whether these effects are conserved across species, we next examined mitochondrial function in *C. elegans*. To specifically evaluate neuronal mitochondria, we utilized transgenic nematodes in which neuronal mitochondria are tagged with an HA epitope, allowing their selective isolation. Seahorse XF analysis revealed that neuronal mitochondria from Ptl1-KO worms exhibited increased basal respiration (Fig. 1 *M* and *N*) and elevated FCCP-stimulated OCR (Fig. 1*P*), together with increased ATP-linked respiration (Fig. 1*Q*) and proton leak (Fig. 1*S*), whereas ADP-stimulated respiration (Fig. 1*O*) and the respiratory control ratio remained unchanged (Fig. 1*R*). These findings demonstrate that loss of Tau/PTL-1 is associated with enhanced mitochondrial respiratory activity in both mouse and nematode neurons.

**Fig. 2.**
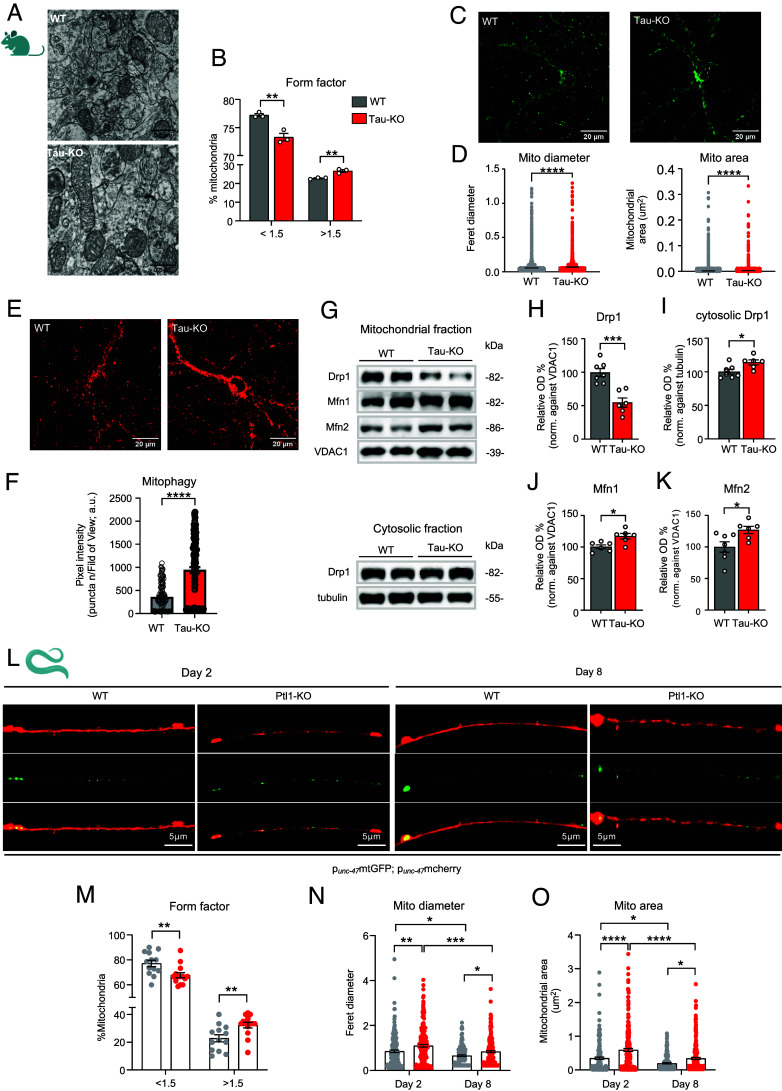
Loss of Tau promotes a pro-fusion mitochondrial state in the mouse brain and *C. elegans* neurons. (*A* and *B*) Representative TEM images of mouse brain mitochondria (*A*; Scale bar, 0.5 μm.) and quantitative analysis of mitochondrial morphology (*B*). Tau-KO mice exhibited a shift toward a more fused mitochondrial network, reflected by a decreased proportion of mitochondria with a form factor <1.5 and an increased proportion with a form factor >1.5 compared to WT animals. Data are presented as mean ± SEM (n = 3; **P < 0.01, two-way ANOVA with Sidak’s multiple comparison test) (*C–F*) Representative confocal images of primary neurons derived from WT and Tau-KO mice. Mitochondrial content was assessed using MitoTracker staining (*C*; Scale bar, 20 μm.), and mitophagy events were detected using the Dojindo Mitophagy Dye (*E*; Scale bar, 20 μm.) under basal conditions. Tau-KO neurons display a more fused mitochondrial network (*D*), reflected by increased mitochondrial diameter and area. In addition, Tau-KO neurons exhibit increased mitophagy events under basal conditions. Data are presented as mean ± SEM, with each data point representing a single mitochondrion (n = 10 images/group for mitophagy analysis and n = 9 to 12 images/group for MitoTracker; ***P < 0.001, unpaired *t* test) (*F*). (*G–K*) Representative immunoblots (*G*) and quantification (*H–K*) of mitochondrial dynamics-related proteins in the mouse brain. Drp1 levels were decreased in the mitochondrial fraction (*H*) and increased in the cytosolic fraction (*I*) in Tau-KO mice, consistent with reduced mitochondrial recruitment of Drp1. In contrast, Mfn1 and Mfn2 levels were increased in the mitochondrial fraction of Tau-KO mice (*J* and *K*), supporting a shift toward enhanced mitochondrial fusion. Data are presented as mean ± SEM (n = 6 to 7 mice/group; *P < 0.05, ***P < 0.001, unpaired *t* test). (*L–O*) Representative confocal images of WT and Ptl1-KO worms expressing GFP-labeled mitochondria in GABAergic neurons (*L*; Scale bar, 5 μm.) At day 8, Ptl1-KO worms displayed a shift toward a more fused mitochondrial network, as indicated by a decreased proportion of mitochondria with a form factor <1.5 and an increased proportion with a form factor >1.5 compared to WT animals (*M*). Consistently, mitochondrial diameter (*N*) and area (*O*) were increased in Ptl1-KO worms. Data are presented as mean ± SEM with each data point representing a single mitochondrion (n = 3; N = 10 to 15 nematodes per group/replicate; *P < 0.05, **P < 0.01, ***P < 0.001, ****P < 0.001, two- way ANOVA with Tukey’s multiple comparison test).

Given the known link between pathological Tau and impaired mitophagy (28), we next examined whether loss of Tau affects mitophagy. Using a dual-fluorescent reporter sensitive to lysosomal pH (GFP/DsRed) (32), we measured mitophagy activity in *C. elegans*. Consistent with previous studies (33), WT worms showed an age-related increase of the GFP/DsRed ratio, reflecting reduced mitophagy efficiency; however, this increase was not detected in Ptl1-KO nematodes (*SI Appendix*, Fig. S2 *A* and *B*). Notably, Ptl1-KO worms displayed significantly enhanced mitophagy compared to WT at day 8 (*SI Appendix*, Fig. S2 *A* and *B*). To further validate the impact of Tau deficiency on mitophagy in a mammalian context, we assessed mitochondrial turnover in primary neurons from Tau-KO and WT littermate mice. Basal mitophagy levels were increased in Tau-KO neurons (Fig. 2 *E* and *F*), consistent with our observations in *C. elegans*. Together, these findings indicate that physiological Tau restrains mitochondrial activity and modulates mitophagy dynamics across species.

### Loss of Tau Induces Pro-Fusion Mitochondria State.

To determine whether the increased mitochondrial activity observed upon Tau loss is associated with alterations in mitochondrial dynamics, we next examined mitochondrial morphology and the expression of key regulators of mitochondrial fusion and fission. TEM analysis of prefrontal brain tissue, focusing on ultrastructural features of mitochondria, revealed a clear shift toward a more fused mitochondrial network in Tau-KO animals (Fig. 2*A*). Quantitative morphometric analysis, using the form factor that incorporates both the shape and the branching of mitochondria (34), showed a decreased proportion of fragmented mitochondria (form factor < 1.5) and a corresponding increase in elongated mitochondria (form factor >1.5) in Tau-KO compared to WT mice, indicating a shift toward a pro-fusion state (Fig. 2*B*). Additionally, primary neurons derived from Tau^−/−^ mice revealed alterations in mitochondrial morphology characterized by elongated mitochondrial network that was characterized by increased mitochondrial diameter and area (Fig. 2 *C* and *D*).

To further characterize mitochondrial dynamics, we examined the levels and subcellular distribution of key regulators of mitochondrial fission and fusion (Fig. 2 *G* and *K* and *SI Appendix*, Fig. S2 *C–E*). Western blot analysis of mitochondrial fraction of mouse revealed a significant reduction of Drp1, a central mediator of mitochondrial fission, in Tau-KO brains compared to WT (Fig. 2*H*), accompanied by a corresponding increase in the cytosolic fraction (Fig. 2*I*). In contrast, no changes were observed in Fis1 levels in the mitochondrial fraction (*SI Appendix*, Fig. S2 *C* and *D*). These findings are consistent with reduced mitochondrial recruitment of Drp1 and suggest a decrease in fission activity. In parallel, we detected elevated levels of the mitochondrial fusion proteins Mitofusin 1 (Mfn1) and Mitofusin 2 (Mfn2) in the mitochondrial fraction of Tau-KO brains compared to WT (Fig. 2 *J* and *K*) supporting a shift toward enhanced mitochondrial fusion. Notably, gene expression analysis revealed no significant differences in the transcript levels of Mfn1, Mfn2, Fis1 accompanied by a reduction of Drp1 mRNA levels consistent with the decreased Drp1 protein levels found in Tau-KOs (*SI Appendix*, Fig. S2*F*).

To determine whether these alterations are conserved across species, we next examined mitochondrial morphology in *C. elegans*. Using transgenic nematodes coexpressing mitochondria-targeted GFP and cytosolic mCherry in GABAergic motor neurons (Fig. 2*L*), we observed a similar shift toward a fused mitochondrial network in Ptl1-KO animals. Specifically, Ptl1-KO worms displayed a decreased proportion of mitochondria with a form factor <1.5 and an increased proportion with a form factor >1.5 compared to WT (Fig. 2*M*). Consistent with these findings, mitochondrial diameter was significantly increased in Ptl1-KO worms at both day 2 and day 8 compared to WT (Fig. 2*N*), accompanied by a corresponding increase in mitochondrial area at day 2 and day 8 (Fig. 2*O*). Additionally, axonal mitochondrial content was increased in GABAergic neurons of Ptl1-KO nematodes compared to WT (*SI Appendix*, Fig. S2*G*). Gene expression analysis in *C. elegans* revealed no significant differences in transcript levels of key mitochondrial dynamics regulators between WT and Ptl1-KO worms (*SI Appendix*, Fig. S2*H*). Furthermore, analysis of endogenous FZO-1::GFP and GFP::DRP-1 reporter strains showed that FZO-1 levels remained unchanged, whereas DRP-1 levels were reduced in Ptl1-KO animals (*SI Appendix*, Fig. S2 *I–L*). To further strengthen these results, we generated transgenic nematodes expressing neuron-specific FZO-1::GFP and GFP::DRP-1 reporters. Quantitative analysis of these reporters in both WT and Ptl1-KO backgrounds confirmed a reduction in DRP-1 levels, while FZO-1 levels remained unaltered (*SI Appendix*, Fig. S2 *M–P*). While these measurements are based on fluorescence intensity and may not fully capture tissue-specific dynamics, they are consistent with reduced fission activity in the absence of PTL-1. Overall, these findings indicate that loss of Tau/PTL-1 promotes a conserved shift toward a pro-fusion mitochondrial state, associated with reduced mitochondrial fission activity and enhanced mitochondrial connectivity in both mice and nematodes.

### Tau Deficiency Promotes Stress Resistance Despite Reduced Baseline Longevity.

**Fig. 3.**
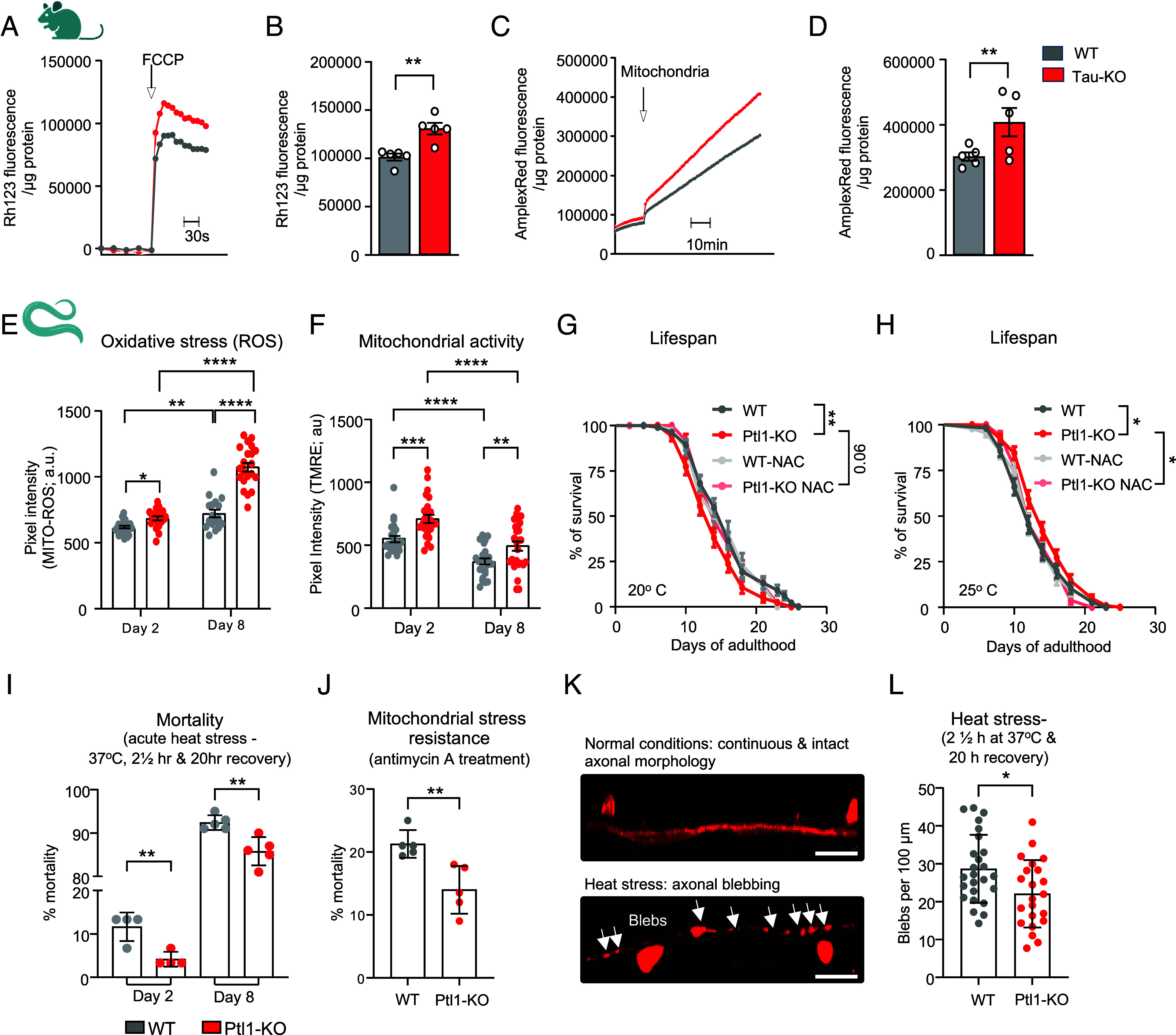
Loss of Tau/PTL-1 is associated with increased mitochondrial membrane potential, elevated ROS levels, and enhanced stress resistance. (*A–D*) Mitochondrial membrane potential and ROS levels were assessed in mitochondria isolated from WT and Tau-KO mouse brain. Tau-KO mitochondria exhibited increased mitochondrial membrane potential (*A* and *B*) and elevated H_2_O_2_ production (*C* and *D*). Panels *A* and *C* show representative real-time traces following sequential addition of assay reagents, while panels *B* and *D* present the corresponding quantification. Data are presented as mean ± SEM (n = 5 mice/group; **P < 0.01, unpaired *t* test). (*E* and *F*) In *C. elegans*, ROS levels measured using MitoSOX (*E*) and mitochondrial membrane potential assessed by TMRE fluorescence (*F*) were increased in Ptl1-KO worms compared to WT at both day 2 and day 8 of adulthood. Data are presented as mean ± SEM (n = 2 to 3 independent experiments; N = 15 to 20 nematodes/group/replicate; **P < 0.01, ***P < 0.001, ****P < 0.001, two-way ANOVA with Tukey’s multiple comparison test). (*G* and *H*) Survival analysis of WT and Ptl1-KO worms under standard growth conditions (20 °C) or in the presence of the antioxidant N-acetylcysteine (NAC), and under mild heat stress (25 °C). Ptl1-KO animals exhibited reduced survival under standard conditions (*G*) but increased survival under mild heat stress compared to WT (*H*). NAC treatment improved survival of Ptl1-KO animals at 20 °C but had no significant effect under heat stress conditions. Survival curves were analyzed by Kaplan–Meier statistical analysis followed by Log-rank/Mantel–Cox test (*SI Appendix*, Table S3) (n = 2 independent experiments; N = 90 to 135 nematodes/group/replicate; *P < 0.05, **P < 0.01). (*I*) Under acute heat stress (37 °C for 2.5 h followed by recovery), Ptl1-KO animals exhibited reduced mortality compared to WT at both ages. Data are presented as mean ± SEM (n = 4 to 5 independent experiments; N = 25 to 30 nematodes/group/replicate; **P < 0.01, two-way ANOVA with Tukey’s multiple comparison test). (*J*) Ptl1-KO animals also showed reduced mortality following mitochondrial stress induced by antimycin A. Data are presented as mean ± SEM (n = 5 independent experiments; N = 25 to 30 nematodes/group/replicate; **P < 0.01, ***P < 0.001, ****P < 0.001, unpaired *t* test). (*K* and *L*) Representative confocal images of axons showing normal morphology (*Upper*) and damaged axons with blebbing (*Lower*) (*K*). Quantification revealed a reduced number of axonal blebs per 100 μm in Ptl1-KO animals compared to WT following acute heat stress (*L*). Data are presented as mean ± SEM, with each data point representing a single nematode (n = 2 independent experiments; N = 20 to 25 nematodes/group/replicate, *P < 0.05, unpaired *t* test).

Given the observed increased bioenergetic activity [increased basal and maximal respiration, as well as ATP production (Fig. 1)], we next examined whether enhanced mitochondrial function is associated with alterations in mitochondrial integrity. Thus, we measured mitochondrial membrane potential and ROS production in mitochondria isolated from Tau-KO and WT mouse brains. Tau-KO samples exhibited increased mitochondrial membrane potential, as reflected by enhanced fluorescence intensity (Fig. 3 *A* and *B*), together with elevated H_2_O_2_ production compared to WT controls (Fig. 3 *C* and *D*), indicating increased mitochondrial activity. We next assessed whether similar changes occur in *C. elegans*. Consistent with the mouse data, Ptl1-KO worms displayed increased mitochondrial ROS levels, (Fig. 3*E*), as well as elevated mitochondrial membrane potential (Fig. 3*F*), at both day 2 and day 8 of adulthood. Although mitochondrial membrane potential declined with age in both genotypes, Ptl1-KO worms consistently maintained a higher mitochondrial membrane potential (Fig. 3*F*), indicating sustained enhancement of mitochondrial activity. These findings indicate that loss of Tau/PTL-1 is associated with enhanced mitochondrial activity across species.

To determine the physiological consequences of increased ROS, we performed lifespan analyses in the presence or absence of the antioxidant NAC. Under standard conditions (20 °C), Ptl1-KO nematodes exhibited reduced lifespan compared to WT (Fig. 3*G*), consistent with a crucial role for PTL-1 in supporting neuronal integrity and organismal homeostasis (35–38). Importantly, NAC supplementation slightly rescued the reduced lifespan of Ptl1-KO worms, restoring survival to levels comparable to WT, while having no detectable effect on WT animals (Fig. 3*G*). In contrast, under mild heat stress (25 °C), which accelerates metabolic and age-related processes, Ptl1-KO nematodes displayed improved survival relative to WT (Fig. 3*H*). Under these conditions, NAC treatment shortened the lifespan of Ptl1-KO worms (Fig. 3*H*), indicating that ROS contributes to the adaptive response observed under stress.

To further assess stress resilience, we exposed nematodes to acute heat shock and mitochondrial stressors. Ptl1-KO worms exhibited significantly reduced mortality following acute heat stress (37 °C, 2.5 h followed by recovery) at both day 2 and day 8 (Fig. 3*I*). Similarly, Ptl1-KO animals showed increased survival following mitochondrial stress induced by antimycin, a complex III inhibitor, (Fig. 3*J*), indicating enhanced resistance to mitochondrial dysfunction. Importantly, this “stress-resistance” phenotype was associated with improved neuronal integrity. Although PTL-1 deficiency has been linked to defective neuronal morphology in touch receptor and GABAergic motor neurons (37, 39), quantification of axonal blebbing in GABAergic motor neurons revealed significantly fewer heat-induced blebs in Ptl1-KO worms compared to WT (Fig. 3 *K* and *L*), indicating improved structural preservation under stress conditions.

Altogether, these findings indicate that loss of Tau/PTL-1 enhances mitochondrial activity and increases ROS production, resulting in a context-dependent physiological outcome. Under basal conditions, elevated ROS contributes to reduced lifespan, which can be reversed by antioxidant treatment. In contrast, under mild stress conditions, ROS supports the adaptive response associated with improved survival, and antioxidant treatment attenuates these beneficial effects. Thus, Tau/PTL-1 acts as a regulator of mitochondrial homeostasis and stress adaptation, balancing bioenergetic activity and oxidative signaling to modulate organismal fitness.

### Conserved Mitochondrial Remodeling Under PTL-1/Tau Deficiency Confers Mitochondrial Fusion-dependent Stress Resilience.

**Fig. 4.**
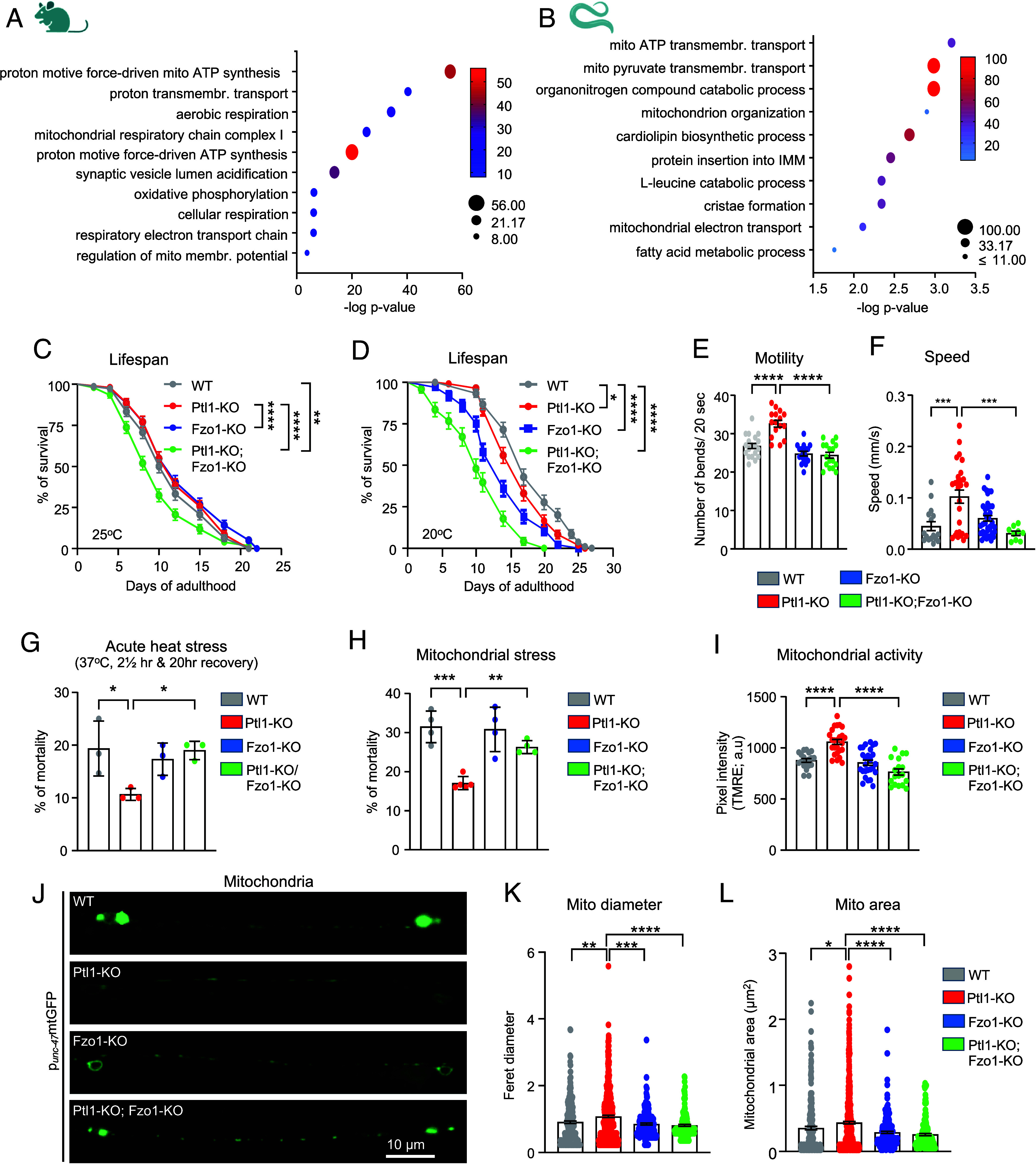
FZO-1 mediates the mitochondrial and stress-resistance phenotypes associated with Tau/PTL-1 loss. (*A* and *B*) GO enrichment analysis of differentially expressed mitochondrial proteins in Tau-KO vs. WT mice (*A*) and Ptl1-KO vs. WT worms (*B*), revealing alterations in pathways related to mitochondrial respiratory complexes, morphology, cristae organization, and metabolic processes. (*C* and *D*) Survival analysis showing that Ptl1-KO/Fzo1-KO double-mutant worms exhibit reduced lifespan compared to Ptl1-KO animals at both 20 °C (*C*) and 25 °C (*D*), indicating that loss of FZO-1 suppresses the longevity phenotype associated with Ptl1 deficiency. Survival curves were analyzed by Kaplan–Meier statistical analysis followed by Log-rank/Mantel–Cox test (*SI Appendix*, Table S3) (n = 2 independent experiments; N = 150 to 160 nematodes/group/replicate; *P < 0.005, **P < 0.01, ****P < 0.0001). (*E* and *F*) Ptl1-KO worms displayed increased motility (*E*) and speed (*F*) compared to WT, whereas these phenotypes were attenuated in Ptl1-KO/Fzo1-KO double mutants, which showed levels comparable to WT animals. Data are presented as mean± SEM, with each data point representing a single nematode (n = 2 to 3 independent experiments; N = 15 to 25 nematodes/group/replicate; *P < 0.05, **P < 0.01, ***P < 0.001, one-way ANOVA with Tukey’s multiple comparison test). (*G* and *H*) The reduced mortality of Ptl1-KO worms under acute heat stress (37 °C) (*G*) and mitochondrial stress induced by antimycin A (*H*) was abolished in Ptl1-KO/Fzo1-KO animals, indicating that FZO-1 is required for the enhanced stress resistance associated with Ptl1 deficiency. Data are presented as mean ± SEM, with each data point representing one replicate (n = 3 to 4 independent experiments; N = 25 to 30 nematodes/group/replicate, *P < 0.05, **P < 0.01, ***P < 0.001, one-way ANOVA with Tukey’s multiple comparison test). (*I*) Mitochondrial membrane potential, assessed by TMRE fluorescence, was elevated in Ptl1-KO worms and restored to WT levels in Ptl1-KO/Fzo1-KO animals. Data are presented as mean ± SEM with each data point representing a single nematode (n = 3 independent experiments; N = 15 to 20 nematodes/group/replicate, ****P < 0.0001, one-way ANOVA with Tukey’s multiple comparison test). (*J–L*) Representative confocal images of axonal mitochondria in WT, Ptl1-KO, Fzo1-KO, and double-mutant worms expressing GFP-labeled mitochondria in GABAergic neurons (*J*; Scale bar, 10 μm.) (*K* and *L*) Ptl1-KO worms exhibited increased mitochondrial area (*K*) and diameter (*L*), indicative of a more fused mitochondrial network. Loss of FZO-1 in the Ptl1-KO background abolished this phenotype, restoring mitochondrial morphology toward WT levels. Data are presented as mean ± SEM, with each data point representing a single mitochondrion (n = 3 independent experiments; N = 10 to 15 nematodes/group/replicate; *P < 0.05, **P < 0.01, ***P < 0.001, ****P < 0.0001, one-way ANOVA with Tukey’s multiple comparison test).

To gain mechanistic insight into the pathways underlying the enhanced mitochondrial activity and stress resistance observed in Tau-KO mice and Ptl1-KO nematodes, we performed quantitative proteomic analyses of hippocampal tissue from WT and Tau-KO mice, as well as whole-body samples from WT and Ptl1-KO worms. We identified 1,596 and 868 significantly differentially expressed proteins in mice and worms, respectively. In both models, enrichment analysis of total proteome (Gene Ontology, GO: Cellular components) revealed mitochondria-related categories among the most significantly affected, supporting a broad remodeling of mitochondrial biology upon Tau/PTL-1 loss. More specifically, GO analysis of differentially expressed mitochondrial proteins uncovered alterations in pathways linked to respiratory complexes, mitochondrial morphology, cristae organization, and metabolic processes (Fig. 4 *A* and *B*), further supporting the notion that Tau/PTL-1 deficiency induces a conserved mitochondrial remodeling program (see also heatmap of up- and down-regulated mitochondrial proteins in *SI Appendix*, Fig. S3 *A* and *B* and Tables S3–S7).

Because both biochemical and imaging data indicated a shift toward a pro-fusion mitochondrial state, we next tested whether mitochondrial fusion is functionally required for the phenotypes associated with PTL-1 deficiency. To this end, we generated double-mutant worms lacking both PTL-1 and FZO-1 [*fzo-1*(*tm1133*)*; ptl-1*(*ok621*), hereafter referred to as Ptl1-KO/Fzo1-KO], as well as double mutants lacking PTL-1 and DRP-1 [*ptl-1*(*ok621*)*; drp-1*(*tm1108*), hereafter referred to as Ptl1-KO/Drp1-KO]. Loss of FZO-1 markedly suppressed the adaptive phenotypes observed in Ptl1-KO worms. Specifically, Ptl1-KO/Fzo1-KO animals exhibited a pronounced reduction in lifespan compared with Ptl1-KO worms under both standard and mild stress conditions (Fig. 4 *C* and *D*). In contrast, DRP-1 deficiency did not significantly alter the lifespan of Ptl1-KO animals at 20 °C and had only modest effects at 25 °C (*SI Appendix*, Fig. S4 *A* and *B*), indicating that loss of DRP-1 does not phenocopy the beneficial effects associated with PTL-1 deficiency. Notably, the motility of *C. elegans* is closely linked to mitochondrial activity, as efficient mitochondrial function is essential for muscle contraction and movement (40). Hence, we next examined locomotor behavior. Ptl1-KO worms displayed enhanced motility and increased speed compared with WT animals (Fig. 4 *E* and *F*). Notably, both phenotypes were suppressed in Ptl1-KO/Fzo1-KO animals, which exhibited values comparable to WT. By contrast, DRP-1 deficiency does not affect the enhanced locomotor phenotype of Ptl1-KO worms (*SI Appendix*, Fig. S4*C*). Similarly, the elevated mitochondrial membrane potential observed in Ptl1-KO animals was abolished in the absence of FZO-1 (Fig. 4*I*). DRP-1 deficiency also normalized TMRE intensity in the Ptl1-KO background (*SI Appendix*, Fig. S4*D*), indicating that both fusion and fission perturbations influence mitochondrial membrane potential, but only FZO-1 loss consistently suppresses the broader adaptive phenotype.

We next assessed whether mitochondrial fusion is required for the enhanced stress resistance of Ptl1-KO worms. Under both acute heat stress and antimycin A-induced mitochondrial stress, Ptl1-KO/Fzo1-KO animals exhibited markedly increased mortality compared with Ptl1-KO worms, thereby abolishing the stress-resistant phenotype associated with PTL-1 deficiency (Fig. 4 *G* and *H*). In contrast, DRP-1 deficiency did not alter resistance to mitochondrial stress in the Ptl1-KO background (*SI Appendix*, Fig. S4*F*), although it did increase sensitivity to acute heat stress (*SI Appendix*, Fig. S4*E*). This observation is consistent with previous reports showing that mitochondrial fission mutants are particularly sensitive to thermal stress (41), likely because acute heat stress activates mitochondrial quality control pathways, including mitophagy, that require intact fission machinery to preserve cellular homeostasis (42, 43). To determine whether these functional changes were associated with altered mitochondrial morphology, we examined mitochondria in GABAergic motor neurons (Fig. 4*J*). As expected, Ptl1-KO worms displayed enlarged and elongated mitochondria, as reflected by increased mitochondrial diameter and area (Fig. 4 *K* and *L*). Importantly, deletion of FZO-1 led to pronounced mitochondrial fragmentation in both WT and Ptl1-KO backgrounds, reversing the pro-fusion phenotype induced by PTL-1 loss (Fig. 4 *K* and *L*). These data demonstrate that FZO-1 is required for the mitochondrial remodeling associated with PTL-1 deficiency.

**Fig. 5.**
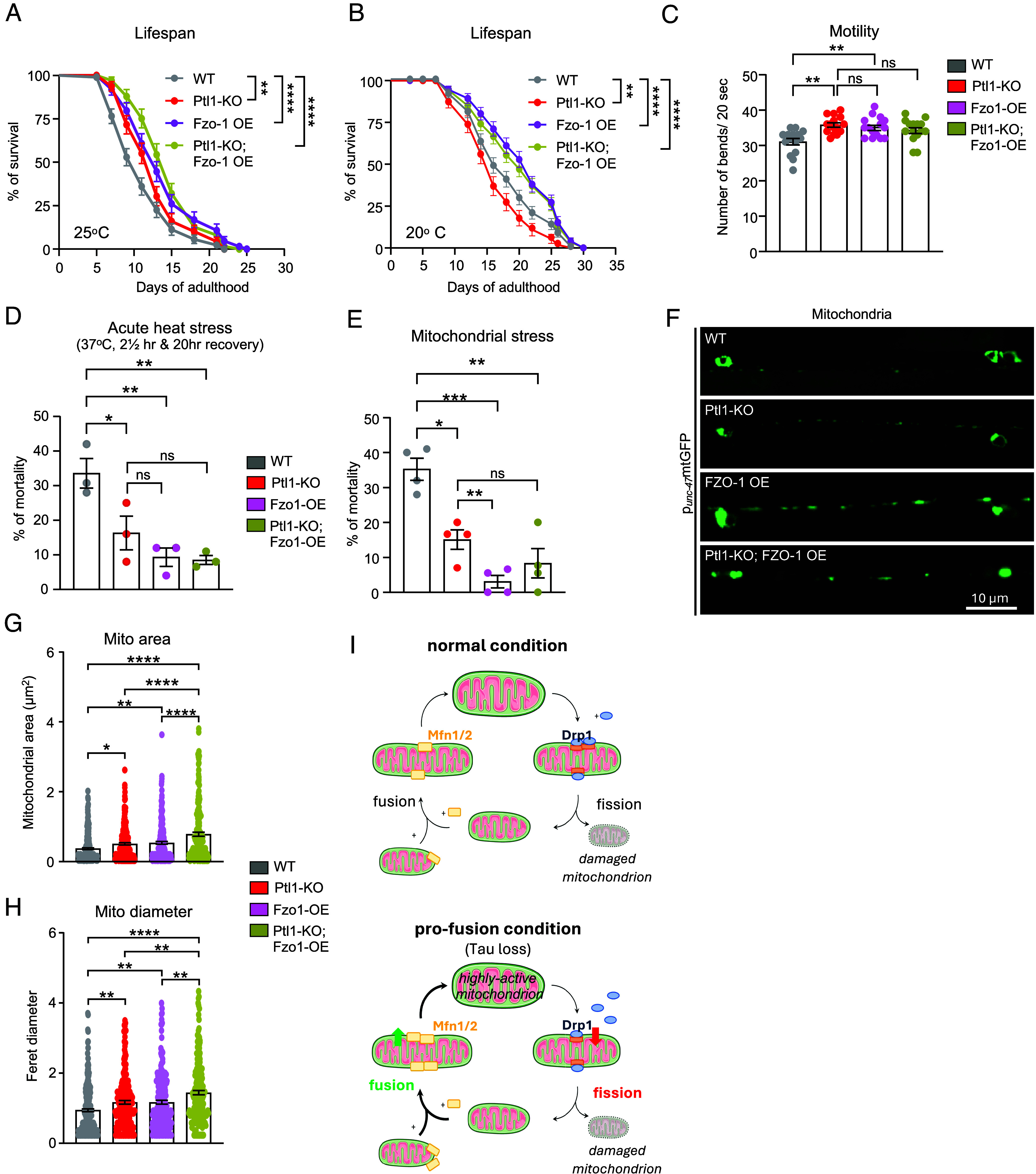
FZO-1 overexpression phenocopies key mitochondrial and stress-resistance features of PTL-1/Tau deficiency. (*A* and *B*) Survival analysis showing that overexpression of FZO-1 (Fzo1-OE) significantly extends lifespan compared to WT animals at both 25 °C (*A*) and 20 °C (*B*). In the Ptl1-KO background, Fzo1-OE further increased lifespan relative to Ptl1-KO worms alone. Survival curves were analyzed by Kaplan–Meier statistical analysis followed by Log-rank/Mantel–Cox test (*SI Appendix*, Table S3) (n = 3 independent experiments; N = 80 to 100 nematodes/group/replicate; **P < 0.01, ****P < 0.0001) (*C*) Fzo1-OE worms displayed increased motility compared to WT animals, whereas Fzo1-OE did not further alter motility in Ptl1-KO worms. Data are presented as mean ± SEM (n = 3 independent experiments; N = 15 to 25 nematodes/group/replicate; ns *P* > 0.05, **P < 0.01, ***P < 0.001, one-way ANOVA with Tukey’s multiple comparison test). (*D* and *E*) Stress resistance assays showing that Fzo1-OE enhanced resistance to acute heat stress (*D*) and mitochondrial stress induced by antimycin A (*E*) compared to WT animals. However, Fzo1-OE did not further increase stress resistance in the Ptl1-KO background. Data are presented as mean ± SEM (n = 3 to 4 independent experiments; N = 25 to 30 nematodes/group/replicate; ns *P* > 0.05, *P < 0.05, **P < 0.01, ***P < 0.001, one-way ANOVA with Tukey’s multiple comparison test). (*F*) Representative confocal images of GFP-labeled mitochondria in GABAergic neurons of WT, Ptl1-KO, Fzo1-OE, and Ptl1-KO/Fzo1-OE worms. (*G* and *H*) Quantification of mitochondrial morphology showing that Fzo1-OE increased mitochondrial area (*G*) and diameter (*H*) compared to WT, consistent with a more fused mitochondrial network similar to that observed in Ptl1-KO worms. In the Ptl1-KO background, Fzo1-OE further increased mitochondrial size parameters. Data are presented as mean ± SEM, with each data point representing a single mitochondrion (n = 3 independent experiments; N = 10 to 15 nematodes/group/replicate; *P < 0.05, **P < 0.01, ****P < 0.0001, one-way ANOVA with Tukey’s multiple comparison test). (*I*) Schematic model summarizing the proposed role of Tau/PTL-1 in regulating mitochondrial function and dynamics where loss of Tau/PTL-1 promotes a pro-fusion state.

To test whether enhanced mitochondrial fusion is not only required but is also sufficient to promote stress resilience, we utilized transgenic nematodes overexpressing FZO-1. FZO-1 overexpression (Fzo1-OE) significantly extended lifespan in WT worms at both 20 °C and 25 °C and further increased lifespan in the Ptl1-KO background (Fig. 5 *A* and *B*). Consistent with a recent study (44), Fzo1-OE alone also promoted increased survival compared with WT animals. Moreover, Fzo1-OE enhanced resistance to both acute heat stress and antimycin A-induced mitochondrial stress (Fig. 5 *D* and *E*) without further increasing motility in Ptl1-KO worms (Fig. 5*C*), suggesting that Fzo1-OE and PTL-1 loss converge on a common adaptive pathway. At the morphological level, Fzo1-OE induced a more fused mitochondrial network in GABAergic motor neurons, characterized by increased mitochondrial area and diameter (Fig. 5 *F*–*H*). In the Ptl1-KO background, Fzo1-OE further increased these parameters, consistent with an additive effect on mitochondrial morphology. Together, these findings indicate that increased mitochondrial fusion is sufficient to phenocopy key cellular and organismal aspects of PTL-1/Tau deficiency.

In parallel, we examined the effects of modulating the fission regulator DRP-1. Overexpression of DRP-1 reduced lifespan in Ptl1-KO animals at both 20 °C and 25 °C (*SI Appendix*, Fig. S4 *G* and *H*), consistent with a shift toward increased mitochondrial fission that is detrimental in the context of PTL-1 deficiency. However, DRP-1 overexpression did not significantly alter motility or resistance to acute heat and mitochondrial stress in the Ptl1-KO background (*SI Appendix*, Fig. S4 *I–K*). Thus, although changes in DRP-1 influence selected physiological outputs, they do not reproduce the broad adaptive phenotype induced by PTL-1 loss.

Taken together, these findings demonstrate that the adaptive phenotypes associated with Tau/PTL-1 deficiency are not passive consequences of altered mitochondrial activity but instead depend on active remodeling of mitochondrial dynamics through FZO-1-mediated fusion. In contrast, modulation of DRP-1 has more limited and context-dependent effects, underscoring mitochondrial fusion as the principal mechanism linking Tau/PTL-1 loss to enhanced mitochondrial function and stress resilience.

## Discussion

While the pathological roles of Tau in neurodegeneration are well established (7–9, 22, 45, 46), the physiological function(s) of WT Tau remains poorly explored. Although several studies have shown that pathological Tau disrupts mitochondrial morphology, dynamics, and mitophagy (11, 32, 47), far less is known about how endogenous Tau regulates mitochondrial homeostasis under physiological conditions. Notably, WT Tau has been detected at both the outer mitochondrial membrane and within mitochondrial compartments (48), suggesting a potential direct role in the regulation of mitochondrial structure and function.

In this context, our findings across *C. elegans* and mouse models revealed a conserved role for WT Tau in fine-tuning mitochondrial dynamics. Specifically, loss of Tau/PTL-1 promotes a shift toward a pro-fusion mitochondrial state, as evidenced by increased mitochondrial elongation in both systems. This is supported by ultrastructural analyses in mouse tissue and high-resolution confocal imaging in primary mouse neurons and worms, indicating enhanced mitochondrial form factor and network connectivity (49, 50). In mammalian neurons, this phenotype is further supported by increased mitochondrial localization of mitofusins and altered DRP-1 distribution, consistent with reduced mitochondrial fission. Importantly, primary neurons derived from Tau-deficient mice also exhibit elongated mitochondrial morphology, reinforcing the relevance of this mechanism in a neuronal context. Together, these structural changes are consistent with the enhanced mitochondrial respiration and ATP production observed upon Tau loss (Fig. 5*I*).

Mitochondrial fusion is widely recognized as a protective mechanism that facilitates the exchange of mitochondrial contents, including proteins, lipids, and mitochondrial DNA, thereby promoting organelle repair, sustaining bioenergetic efficiency, and buffering metabolic stress (51–53). In contrast, mitochondrial fission is essential for quality control, enabling the segregation and removal of damaged mitochondrial components and supporting metabolic flexibility under stress. Disruption of mitochondrial dynamics, including reduced mitofusin activity or excessive DRP1-driven fission, has been implicated in multiple neurodegenerative disorders (54–56), underscoring the critical role of mitochondrial architecture in neuronal integrity.

Previous studies have shown a direct link between mitofusin activity and mitochondrial bioenergetics. Enhanced MFN1/2 function promotes mitochondrial elongation, which is associated with increased membrane potential, respiratory capacity, and ATP production (57). Conversely, inhibition or loss of mitofusins compromises mitochondrial function (57–59). Although the precise molecular coupling between mitochondrial morphology and function remains incompletely defined, mitochondrial quality control pathways likely contribute to this relationship. In this regard, our data indicate that Tau/PTL-1 deficiency is associated with a dynamic regulation of mitophagy, consistent with a fusion-favoring mitochondrial network that modulates mitochondrial turnover in a context-dependent manner.

In addition to its role in maintaining bioenergetics, mitochondrial fusion contributes to cellular resilience under stress by enabling content mixing between mitochondria, thereby diluting damage and preserving function (60). Moreover, inhibition of mitochondrial fission and promoting fusion has been shown to enhance cellular resilience to thermal stress (61). Consistently, we observed that PTL-1/Tau deficient worms display enhanced resistance to both thermal and mitochondrial stress, while maintaining neuronal integrity under challenged conditions. Notably, this stress-resistant phenotype is abolished upon genetic ablation of FZO-1, demonstrating that mitochondrial fusion is required for the adaptive response induced by PTL-1/Tau loss. While mitofusins are also known to regulate ER–mitochondria contacts, which can influence calcium signaling and metabolic coordination (62), direct assessment of these interactions needs to be further investigated in future studies. Nevertheless, it is plausible that enhanced interorganellar communication contributes to the improved mitochondrial performance and stress resilience observed in Tau/PTL-1–deficient animals.

Tau has been extensively studied in the context of its toxic gain-of-function effects in AD and related tauopathies. However, the consequences of losing normal Tau function remain less clear and, in some cases, controversial. While several studies report minimal effects on brain physiology or behavior, others describe subtle deficits (6, 16, 37, 46, 63–65). Derived from two evolutionary distant organisms, our findings demonstrate that Tau loss induces a conserved shift toward mitochondrial fusion and enhanced mitochondrial bioenergetics, revealing a physiological role for Tau in restraining mitochondrial dynamics. In contrast, overexpression of WT or mutant Tau in cellular and animal models promotes mitochondrial fragmentation, impairs mitochondrial function and disrupts mitophagy (11, 28, 29, 66), suggesting that excessive Tau exerts an inhibitory effect on mitochondrial homeostasis. Given that mitochondrial damage and impaired mitophagy are key hallmarks of aging and neurodegeneration (67–70), our findings raise the possibility that Tau-lowering strategies, such as antisense oligonucleotides currently under investigation for AD, may exert beneficial effects, at least in part, by relieving Tau-mediated constraints on mitochondrial fusion and function. Importantly, our data also indicate that the consequences of Tau loss are context-dependent, as enhanced mitochondrial activity is associated with both increased ROS production and improved stress resilience, highlighting a balance between metabolic activation and adaptive responses.

Taken together, our study uncovers a previously underappreciated role for WT Tau in the regulation of mitochondrial morphology, bioenergetics, and stress adaptation. These findings extend the functional landscape of Tau beyond its pathological roles and identify mitochondrial dynamics, particularly fusion, as a central mechanism linking Tau to cellular homeostasis. Future studies should further define the molecular interfaces between Tau and mitochondrial dynamics machinery and determine how these interactions affect susceptibility to neurodegenerative and stress-related disorders. Notably, emerging evidence implicates Tau in a broader range of neurological conditions, including stress-related depression (16, 71), epilepsy (72), traumatic brain injury (73), and chronic pain (74), highlighting the wider relevance of Tau-mediated mitochondrial regulation in brain health.

## Supplementary Material

Appendix 01 (PDF)

## Data Availability

All study data are included in the article and/or *SI* *Appendix*.
