# Supplementary material for: Tau protein as a regulator of mitochondrial function and dynamics
Source: Proc Natl Acad Sci U S A. 2026 Jun 30;123(27):e2521642123. doi: 10.1073/pnas.2521642123 (PMC13342828; doi:10.1073/pnas.2521642123)
Supplement: Supplementary file 1 — Appendix 01 (PDF) [file pnas.2521642123.sapp.pdf]

PNAS

Supporting Information for

Tau protein as a regulator of mitochondrial function and dynamics

Eleni Tsakiri[1], Carlos Campos-Marques[2,3], Christina Ploumi[1], Kalliopi Skourti[4], Antonis Roussos[1], Eirini Mytilinaiou[1], Anastasia Vamvaka Iakovou[4], Ildete Luísa Ferreira[5,10], Chrysoula Dioli[2,3,4], Despoina D. Gianniou[13], Martina Samiotaki[7], Jonas Campos[2,3], Clarissa Waites[8,9], Nuno Sousa[11,12], Ioannis Trougakos[13], Joana M Silva[2,3], A. Cristina Rego[5,6], Ioannis Sotiropoulos[2,3,4*] and Konstantinos Palikaras[1*]

[1] Department of Physiology, Medical School, National and Kapodistrian University of Athens, 11527, Athens, Greece
[2] Life and Health Sciences Research Institute (ICVS), University of Minho, Braga, Portugal
[3] ICVS/3B's - PT Government Associate Laboratory, Braga/Guimarães, Portugal
[4] Institute of Biosciences and Applications, National Centre for Scientific Research (NCSR) Demokritos, Agia Paraskevi, Greece
[5]CNC -UC- Center for Neuroscience and Cell Biology, University of Coimbra, Coimbra, Portugal; CIBB - Center for Innovative Biomedicine and Biotechnology, University of Coimbra, Coimbra, Portugal
[6]FMUC - Faculty of Medicine, University of Coimbra, 3000-354 Coimbra, Portugal
[7] Biomedical Sciences Research Center "Alexander Fleming", Institute for Bio-innovation, 16672 Vari, Greece.
[8] Department of Pathology and Cell Biology, Taub Institute for Research on Alzheimer's Disease and Aging Brain, Columbia University Irving Medical Center, New York, NY 10032, USA.
[9] Department of Neuroscience, Columbia University, New York, NY 10032, USA.
[10] III-UC – Institute of Interdisciplinary Research, University of Coimbra, 3030-789 Coimbra, Portugal.
[11] Centro Universitário de Jaguariúna (UniFAJ), São Paulo, Brazil.
[12] Centro Universitário Max-Planck (UniMAX), São Paulo, Brazil.
[13] Department of Biology, National and Kapodistrian University of Athens, 15784, Athens, Greece

*Corresponding authors: Ioannis Sotiropoulos and Konstantinos Palikaras

Email: ioannis@bio.demokritos.gr and palikarask@med.uoa.gr

This PDF file includes:

Supporting Text
Figures S1 to S4
Tables S1 to S7
Material and methods
SI References

Supporting Information Text

Materials and Methods

***C. elegans* strains.** Standard *C. elegans* strain maintenance procedures were followed. The nematode rearing temperature was 20°C or 25ºC for all used strains. These strains were maintained in 60-mm Petri dishes with NGM-agar seeded with *Escherichia coli* OP50. All *C. elegans* strains used in this study are shown in *SI Appendix*, Table S1.

Nematode strain generation. To generate the transgenic animals expressing neuron-specific GFP::DRP-1 and FZO-1::GFP, we used DNA microinjection in the syncytium region of *C. elegans* germlines. Wild-type (N2) young day-1 adult animals were injected with a mix containing the plasmid [p_{rgef-1}GFP::DRP-1] or [p_{rgef-1}FZO-1::GFP] and the selection marker plasmid pRF4, at a final concentration of 25 ng/μL each.

For generating the GFP::DRP-1 expressing vector, we amplified the *drp-1* gene including its stop codon from genomic DNA with the following primers that include EcoRV restriction sites: EcoRV_FW 5'-GATATCATGGAAAATCTCATTCCTGTCGTC-3' and EcoRV_RV 5'-GATATCTCACCAAACTTGTGTTTCTCTCAC-3'. The 2,654-bp PCR product was first cloned into the pCR-II TOPO vector and subsequently subcloned into the pPD96.41 vector. The pPD96.41 vector, which already contained the *rgef-1* promoter and a GFP sequence lacking a stop codon, was pre-digested with EcoRV and dephosphorylated prior to ligation. The insert was cloned downstream of the GFP sequence after digestion with EcoRV.

For generating the FZO-1::GFP expressing vector, we amplified the *fzo-1* gene without stop codon from genomic DNA with the following primers that include BamHI and KpnI restriction sites, respectively: BamHI_FW 5'-GGATCCATGTCTGGCACAGCAAGCTTAGTTC-3' and CC_KpnI_RV '5-GGTACCGGTGGCGTTGGCGGAGAGTCCGATC-3'. The 2,641-bp PCR product was first cloned into the pCR-II TOPO vector and then subcloned into the pPD95.77 vector. Both the insert and the pPD95.77 vector were digested with BamHI and KpnI prior to ligation. The pPD95.77 vector already contained the *rgef-1* promoter.

Mouse lines. Tau-knock out (Tau-KO) and their wild type (WT) littermates mice (C57BL/6J background; 4-5 month-old males) were used in this study (1). All experiments were conducted in accordance with the Portuguese national authority for animal experimentation, *Direcção Geral de Veterinária* (ID: DGV9457). Animals were kept and handled in accordance with the guidelines for the care and handling of laboratory animals in the Directive 2010/63/EU of the European Parliament and Council. Mice were housed in groups of 4-5 per cage under standard environmental conditions (lights on from 8 a.m. [ZT0] to 8 p.m. [ZT12]; room temperature 22ºC; relative humidity of 55%, *ad libitum* access to food and water).

Chemicals. Adenosine 5' diphosphate (ADP) potassium salt, antimycin A, carbonyl cyanide-4-(trifluoromethoxy)phenylhydrazone (FCCP), L-malic acid, L-ascorbic acid, oligomycin, polyethyleneimine (PEI), rotenone, sodium pyruvate and succinic acid were from Sigma Chemical Co. (St Louis, MO, USA). All other reagents were of analytical grade.

Locomotion assessment. *Thrashing assay:* To assess thrashing, 10–15 µL of M9 buffer was pipetted onto a glass slide, and individual worms were transferred into the buffer. Thrashing was quantified by counting body bends, with a complete left-right motion defined as one body bend, over a 20-second period. A total of 15 worms were analyzed per strain, with body bends counted twice for each worm, from three independent experiments. The average body bend count for each worm was used for analysis.

Video recording-based locomotion: Locomotion was recorded using the WMicrotracker® SMART-8x Carousel version (Phylumtech) on unseeded 35 mm NGM plates. Synchronized day 2 adult worms were recorded for 5 minutes. A total of 20-25 worms per strain were examined across two independent experiments. Movement data, including speed (mm/s), was exported to Excel for quantitative analysis.

Mitophagy and mitochondrial morphology assessment in worms. Nematodes were immobilized in a 40 mM tetramisole/M9 buffer drop on microscopic slides, sealed with coverslips and analyzed with the EVOS M7000 system or Zeiss LSM 900 confocal microscope (for mitophagy assessment and mitochondrial morphology). Quantification of the mean pixel intensity was performed by using the Fiji software. Mitophagy was assessed by measuring the ratio GFP to DsRed fluorescent intensity. A total of 10-25 worms per strain were examined across three independent experiments. Statistical analysis was performed by two-way ANOVA with Tukey's multiple comparison test.

Imaging analysis of FZO-1::GFP and GFP::DRP-1 nematode strains. Nematodes were immobilized in a 40 mM tetramisole solution prepared in M9 buffer, mounted on microscope slides, and sealed with coverslips. The *C. elegans* strains *fzo-1(zju136*[FZO-1::GFP]*)* and *drp-1*(*or1941*[GFP::DRP-1]) were imaged using the EVOS M7000 imaging system, whereas neuron-specific transgenic strains were analyzed using the Zeiss LSM 900 confocal microscope. Mean pixel intensity was quantified using Fiji software. For each strain, 10-25 worms were analyzed across 2-3 independent experiments. Statistical significance was assessed using an unpaired t-test.

***C. elegans* lifespan assays.** Lifespan assays were performed at 25°C to simulate mild heat stress. Worms were transferred to freshly OP50-seeded NGM plates daily for the first week, then every

other day thereafter to ensure a consistent food source and avoid contamination. Worms that exhibited desiccation on the plate edge, bagging due to internal hatching, vulvar ruptures, or were otherwise missing were censored from the analysis to maintain data integrity. For each experimental condition, more than 150 worms were distributed across five plates, with at least 20 worms per plate, unless specified otherwise. Lifespan data were analyzed using Kaplan-Meier survival analysis and visualized with GraphPad Prism. This statistical approach provided insights into survival trends and comparisons between experimental conditions. The statistical analysis is shown in *SI Appendix*, Table S1.

RNA isolation and RT-qPCR in *C. elegans*. *C. elegans* total RNA was prepared from frozen worm pellets of OP50-fed WT and Ptl1-KO worms using Nucleozol (Macherey-Nagel). The quality and quantity of RNA samples were determined using BioTek Cytation 5 reader (Agilent). Reverse transcription was carried out with iScript RT cDNA Synthesis KIT (Bio-Rad) and quantitative PCR was performed using KAPA SYBRFAST Universal Kit (Kapa Biosystems) in the QuantStudio 5 Real-Time PCR system (Applied Biosystems). Relative amounts of mRNA were determined using the comparative Ct method and each sample was independently normalized to its endogenous reference gene (*ama-1*). A total of 150-200 worms per strain were examined in two independent experiments. Gene expression data are presented as the mean fold change ± SEM of all biological replicates relative to the indicated control. Statistical analysis was performed using an unpaired t-test. Primers used for qRT-PCR are listed in *SI Appendix*, Table S2.

Mitochondrial membrane potential and ROS measurement in *C. elegans*. Age-synchronized worms were incubated overnight on OP50-seeded NGM plates supplemented with 150 nM tetramethylrhodamine ethyl ester (TMRE; T669, Thermo Fisher Scientific) or 5 μM MitoTracker Red CMXros (M7512, Thermo Fisher Scientific) to stain mitochondrial membrane potential and reactive oxygen species (ROS), respectively. Following staining, worms were transferred to glass slides and immobilized using 40 mM tetramisole to ensure stability during imaging. Slides were examined using the EVOS M7000 imaging system (Thermo Fisher Scientific) to capture fluorescence signals. Fluorescence intensity was quantitatively analyzed using Fiji software, enabling precise pixel intensity measurements to assess mitochondrial potential. A total of 15–20 worms per strain were examined in three independent experiments. Statistical analysis was performed by two-way ANOVA with Tukey’s multiple comparison test.

Stress assays. *Heat Stress Assay*: Worms were incubated on OP50-seeded NGM plates for 2 hours and 30 minutes under heat stress conditions. Following a 20-hour recovery period, worms were evaluated for viability, and dead worms were scored. Surviving worms were either scored or used for imaging analysis to assess physiological or molecular responses. A total of 25-30 worms

per strain were examined in three or four independent experiments. Statistical analysis was performed by two-way ANOVA with Tukey's multiple comparison test.

Mitochondrial Stress Assay: Antimycin was prepared fresh for each experiment. A small volume (100 µL) of the antimycin solution was added to plates to achieve the desired final concentration relative to the total media volume. Plates were overlaid with the drug solution and allowed to equilibrate with the agar medium for 30 minutes to 1 hour prior to use, ensuring uniform drug distribution. Worms were incubated on OP50-seeded NGM plates supplemented with 5 µM antimycin for 24 hours. After the incubation period, worms' viability was assessed by scoring dead worms. A total of 25-30 worms per strain were examined in three or four independent experiments. Statistical analysis was performed by two-way ANOVA with Tukey's multiple comparison test or using an unpaired t-test (as indicated in each figure legend).

Mitochondrial isolation and oxygen consumption rate evaluation by Seahorse analyser in worms. Mitochondria were isolated from large populations of *C. elegans* as described in Ahier et al., 2018 with minor modifications (2). Briefly, 50,000 worms at day 2 were harvested and washed three times with M9 buffer, followed by one wash with ddH_2O. Worm pellets were resuspended in 3 mL ice-cold mitochondrial isolation buffer (MIB) (50 mM KCl, 110 mM mannitol, 70 mM sucrose, 0.1 mM EDTA pH 8.0, 5 mM Tris-HCl pH 7.4, protease inhibitors) and homogenized on ice using a Dounce homogenizer (21 gentle strokes). Homogenates were centrifuged sequentially at 200g (5 min) and 800g (10 min) to remove debris, followed by 12,000g (10 min) to pellet total mitochondria. Mitochondrial pellets were resuspended in 500 µL PEB buffer (PBS, 2 mM EDTA, 1% BSA) and incubated with 100 µg anti-HA magnetic beads (Cat. No. 88836, Thermo Scientific^{TM}) at 4°C for 1 h with rotation. Bead-bound mitochondria were isolated using a magnetic column setup and washed with ice-cold PEB. Mitochondria were finally eluted in 100 µl MIB for each sample.

Purified mitochondria were diluted in 100 µL MIB, and 80 µL from each sample was subjected to bioenergetic profiling using the Seahorse XF24 platform, with 40 µL plated per well. To ensure mitochondrial adherence, assay plates were centrifuged at 2,200 x g for 5 min at 4°C and then equilibrated for 20 min at 20°C before OCR acquisition. Baseline respiration was measured in assay medium containing glutamate (5 mM; G1251, Sigma)) and malate (2.5 mM; W237418, Sigma). State 3 respiration was initiated by ADP (4 mM; 01905, Sigma), whereas ATP synthase-dependent respiration was blocked by oligomycin (2.5 µg/mL; O4876, Sigma). FCCP (4 µM; 75351, Cayman Chemical) was then applied to determine maximal respiratory capacity. Residual respiration was measured after combined inhibition with antimycin A (4 µM; A8674, Sigma) and rotenone (2 µM; R8875, Sigma). Data derive from three independent experiments with two technical replicates per biological replicate, and were analyzed using an unpaired t test.

Mitochondrial isolation by percoll gradient in mice. Macro-dissected prefrontal cortex of mouse brain (n=10-12/group) was washed in ice-cold isolation buffer containing 225 mM mannitol, 75 mM sucrose, 1 mM EGTA, 5 mM HEPES, pH 7.2/KOH, and mitochondria were then isolated using discontinuous percoll density gradient centrifugation as previously described (3). Tissues were homogenized in Dounce All-Glass Tissue Grinder (Kontes Glass Co., Vineland, NJ, USA) and after a brief centrifugation at 1100*g* for 2 min, at 4°C, the supernatant was mixed with freshly made 80% Percoll prepared in 1 M sucrose, 50 mM HEPES, 10 mM EGTA, pH 7.0, then carefully layered on the top of freshly made 10% Percoll (prepared from 80% Percoll) and further centrifuged at 18,500*g* for 10 min, at 4°C. Mitochondria enriched pellet was resuspended in 1 mL of washing buffer containing 250 mM sucrose, 5 mM HEPES-KOH, 0.1 mM EGTA, pH 7.2, and centrifuged again at 10,000*g* for 5 min, at 4°C. Finally, the mitochondrial pellet was resuspended in ice-cold washing buffer and the amount of protein quantified by the Bio-Rad protein assay. Isolated mitochondria were kept on ice until use for further functional analysis. Alternatively, samples were frozen at -80°C.

Oxygen consumption rate evaluation by Seahorse analyser in mice. Oxygen consumption rate (OCR) was determined in freshly isolated prefrontal cortical mitochondria (5 µg; n = 10–12 mice per group) using a Seahorse XF24 Extracellular Flux Analyzer, as previously described (3). Mitochondria were seeded onto polyethyleneimine-coated XF24 plates (1:15,000 dilution) in mitochondrial assay solution (MAS) and centrifuged at 2,200 x *g* for 20 min at 4°C to facilitate adherence. Attachment efficiency was verified by light microscopy (20X objective). Following an equilibration step of 8 min at 37°C, OCR measurements were initiated.

Two experimental protocols were applied (see *SI Appendix*, Fig. S1*B*): (i) assessment of respiratory coupling and (ii) evaluation of electron transport chain (ETC) activity across complexes I-IV. For coupling experiments, basal respiration was measured in MAS containing succinate (10 mM) in the presence of rotenone (2 µM) to inhibit complex I. ADP (4 mM) was then added to stimulate oxidative phosphorylation, followed by oligomycin (2.5 µg/mL) to inhibit ATP synthase. Maximal respiratory capacity was determined after addition of FCCP (4 µM), and residual OCR was obtained following inhibition with antimycin A (4 µM).

For electron flow analysis, mitochondria were assayed under uncoupled conditions in MAS supplemented with FCCP (4 µM), pyruvate (10 mM), and malate (2 mM) to drive substrate-dependent respiration. Rotenone (2 µM) was used to block complex I activity, while subsequent addition of succinate (10 mM) enabled respiration via complex II. Inhibition of complex III with antimycin A (4 µM) suppressed OCR, and complex IV activity was assessed by addition of ascorbate (10 mM) and TMPD (100 µM), which donate electrons directly to cytochrome c, resulting in increased oxygen consumption (*SI Appendix*, Fig. S1*B*).

Mitochondrial membrane potential measurement in mice. Freshly isolated mitochondria (5 µg) were resuspended in K+-based medium (125 mM KCl, 0.5 mM $MgCl_2$, 3 mM KH_2PO_4, 0.01 mM EGTA, 10 mM HEPES; pH 7.4) supplemented with 3 mM succinate, 3 mM glutamate and 0.1 mM of ADP, containing 250 nM Rh123, and incubated at 30°C for 30 min. Fluorescence was recorded at 500 nm excitation and 540 nm emission using a SpectraMax iD3 (Molecular Devices). Following a 2 min baseline, 2.5 µM FCCP was added to induce maximal mitochondrial depolarization and the response recorded for an additional 2 min. Statistical analysis was performed using an unpaired t-test.

Hydrogen peroxide levels determination in mice. Freshly isolated mitochondria (5 µg) were added to K+-based medium (125 mM KCl, 0.5 mM $MgCl_2$, 3 mM KH_2PO_4, 0.01 mM EGTA, 10 mM HEPES; pH 7.4) supplemented with 3 mM succinate, 3 mM glutamate and 0.1 mM of ADP, containing 10 µM AmplexRed plus 0.5 units/mL horseradish peroxidase (HRP), and fluorescence measured for 1 h at 30°C after a 10 min baseline (in the absence of mitochondria) at 571 nm excitation and 585 nm emission using a SpectraMax iD3 (Molecular Devices). Statistical analysis was performed using an unpaired t-test.

Mitochondria analysis by transmission electron microscopy (TEM) in mice. Tau-KO and WT mice (n=3/group) were transcranially perfused as previously described (4) [4% PFA in microtubule stabilization buffer (65 mM PIPES, 25 mM HEPES, 10 mM EGTA, 3 mM $MgCl_2$, pH=6.9]. Next, mouse brains were post-fixed overnight at room temperature in 4% PFA, 0.8% glutaraldehyde in microtubule stabilization buffer. At the next day, brains were incubated to 4% PFA, 0.8 % glutaraldehyde in 0.1 M of phosphate buffer (pH 7.4) for 2 hours and then, transferred to 0.1 M phosphate buffer (4°C). The medial prefrontal cortex brain area was dissected from vibratome coronal sections (300 µm). Sections were embedded in Epon resin along the superficial-to-deep axis and ultrathin sections (500 Å) were cut onto nickel grids. Approx. 100 TEM (30000x) non-overlapping images per group of counterstained ultrathin-sections were obtained using JEM-1400 transmission electron microscope (JEOL, Tokyo, Japan) and Orious Sc1000 digital camera and were analyzed by an experimenter blind to the sample source.

Neuronal primary cultures from mice. For primary neuronal cultures, 24-well plates were coated overnight at 37°C, 5% CO_2, with poly-ornithine (Sigma-Aldrich, #P3655-10MG) in Borate buffer 0.1M pH 8.5. The following day, poly-ornithine was removed, and plating medium [DMEM; 10% FBS; 1% GlutaMAX (Gibco, #35050-038); 0.5% pen/strep (ThermoFisher, #15140122); 0.8% MEM vitamins (ThermoFisher, #11120037)] was added. Plates were kept at 37°C, 5% CO2. Primary neurons were obtained from Tau-KO mice and WT littermates. Mouse pups were sacrificed at P0-P1, and brains were collected to Petri dishes containing cold HBSS solution [HBSS (ThermoFisher,

#14175053); 1% HEPES (ThermoFisher, #15630056); 1% pyruvic acid (ThermoFisher, #11360039); 0.5% pen/strep]. Cortices were dissected, and meninges were removed. Tissue was transferred to new HBSS-containing dishes, minced into small pieces, and collected into a 15 mL conical tube using a sterile Pasteur pipette. After two washes with HBSS at room temperature (RT), 500 µL of trypsin 2.5% (ThermoFisher, #15090046) in 4.5 mL of HBSS was added and incubated for 25 min at 37ºC. Subsequently, 20 µL of DNase-I (Sigma-Aldrich, #D5025-150KU) was added and mixed for 1 min at RT, followed by three washes with HBSS. Following the addition of 4 mL plating medium, tissue was homogenized by pipetting up and down several times using a P1000 pipette, and then centrifuged at 900g for 4 min at RT. The resulting pellet was resuspended in 1 mL plating medium, and cells were seeded at approximately 150.000 cells per well. After 2h at 37°C, medium was replaced with neuronal primary culture medium [Neurobasal A (ThermoFisher, #10888022); 1% GlutaMAX; 2% B-27 supplement (ThermoFisher, #17504044)], and cells were kept at 37°C, 5% CO2. After 15 days in culture, primary neuronal cultures were incubated with either 200 nM MitoTracker™ Green FM (Invitrogen, #M7514) or 100 nM Mitophagy Dye (Dojindo, #MT02) for 45 min. Widefield fluorescent images were acquired using a 40X objective (NA 0.60), imaging from 7-12 wells per genotype/dye. Images were taken from neuronal enriched areas to normalize for differences in cell confluence between genotypes. Image analysis (9-12 images/group) was performed using Fiji (NIH). Average fluorescence intensity was quantified after automatic global thresholding (5, 6). Puncta were counted following Gaussian Blur filtering (sigma=2) and analysis with "Find Maxima" function (prominence=6). Statistical analysis was performed using an unpaired t-test.

Western blot and qPCR analysis in mice. For Western Blot analysis mouse brain tissue (n=6-7/group) was homogenized [250 mM Sucrose, 20 mM HEPES, 10 mM KCl, 1.5 mM $MgCl_2$, 1 mM EDTA, 1 mM EGTA, pH 7.5, 1 mM DTT, Proteinase Inhibitor Cocktail and Phosphatase Inhibitor Cocktails II and III]. After centrifugations [2300 rpm, 12 min & 10600 rpm, 20 min, 4°C], the pellet (mitochondrial fraction) and supernatant (cytosolic fraction) was separated and diluted in TNC buffer [10 mM Tris Acetate pH 8.0, 0.5% Nonidet P40, 5 mM $CaCl_2$, Proteinase Inhibitor Cocktail and Phosphatase Inhibitor Cocktails II and III]. The fractionated samples were electrophoresed and semi-dry transferred onto nitrocellulose membranes (*Trans-Blot® Turbo^{TM} Blotting System, BioRad*). Next, membranes were blocked with milk 5% in TBS-T for 1h. Primary antibodies were diluted in milk 2.5% in TBS-T and incubated with the membranes overnight at 4°C. The primary antibodies used were: Drp1 (1:1000, Abcam); GAPDH (1:5000, Abcam); Mfn2 (1:1000, Sigma); Hsp60 (1:500, Abcam); VDAC1 (1:2000, Abcam). Next, the membranes were incubated for 2 h with the appropriate secondary antibodies and signal quantification was achieved using a ChemiDoc instrument and ImageLab software from Bio-Rad. All values were normalized and expressed as a percentage of control values. Statistical analysis was performed using an unpaired t-test.

For qPCR analysis, total RNA was isolated from mouse hippocampus (4-5 animals/group) using TRIzol Reagent (Invitrogen, #15596026) according to the manufacturer's instructions. RNA concentration and purity were assessed using NanoDrop spectrophotometer (Thermo Fisher Scientific). cDNA was synthesized from 1 µg of total RNA using NZY First-Strand cDNA Synthesis Flexible Pack (NZYtech, #MB40001), following the manufacturer's protocol. Quantitative PCR was performed using SsoFast EvaGreen Supermix (Bio-Rad, #1725202) on 7500 Fast Real-Time PCR System (Applied Biosystems). Each reaction (final volume 10 µL) contained 5 µL master mix, primer concentration 500 nM each, and 1 µL cDNA. The thermocycling conditions were as follows: initial denaturation at 95°C for 10 minutes, followed by 40 cycles of denaturation at 95°C for 15 seconds, and annealing at 60°C for 1 minute. All reactions were performed in triplicates with 4-5 independent biological replicates. Gene expression levels were normalized to B2m (housekeeping gene), and relative expression was calculated using the 2(–ΔΔCt) method. Statistical analysis was performed using an unpaired t-test. Primers used for qRT-PCR are listed in *SI Appendix*, Table S2.

Proteomic analysis in worms and mice. Groups of wild-type (WT) and Ptl1-KO [*ptl-1(ok621)*] worms (day 2) and 4-5 month-old male WT and Tau-KO mice (N=3-4 per group) were compared at their proteome levels through quantitative proteomics using LC-MS/MS methodology. Initially, the worms were collected in three biological replicates per genotype and processed in order to extract their proteins at a whole proteome level by subjecting the individuals to homogenisation and complete lysis in 4%SDS and 100 mM DTT assisted by a heating (99ºC; 5min) and sonication. Homogenates were centrifuged at 17000x g for 15 minutes and the supernatants/protein extracts collected and processed according to the Sp3 protocol (34). For the mouse proteomic analysis, hippocampal tissue was homogenized in chilled RIPA 1 buffer [50 mM Tris-HCl; pH 7.4, 150 mM NaCl, 1 mM EDTA, Complete Protease Inhibitor (Roche), and Phosphatase Inhibitor Mixtures II and III (Sigma)], and centrifuged (20.000 g, 20 min, 4ºC). The resulting supernatant was mixed 1:1 (v/v) with RIPA 2 buffer [50 mM Tris-HCl; pH 8, 150 mM NaCl, 1% NP-40, 0.1% SDS, 0.5 mM sodium deoxycholate, 1x Complete Protease Inhibitor (Roche), and 1x Phosphatase Inhibitor mixes II and III (Sigma), 2% Triton X-100], and the mixture was ultracentrifuged (100000g, 1h, 4ºC). The resulting supernatant (soluble fraction) was collected and processed using the Sp3 protocol. The Sp3 protocol results in the tryptic digestion of the proteins, that after a Sp3 mediated peptide clean-up are ready to be analyzed through LC-MS/MS operating in DIA mode. Our system consists of a Dionex Ultimate 3000RSLC online with a Thermo Q Exactive HF-X Orbitrap mass spectrometer. The peptidic samples were directly injected and separated on an 25 cm-long analytical C18 column (PepSep, 1.9µm^3 beads, 75 µm ID) using an one-hour long run, starting with a gradient of 7% Buffer B (0.1% Formic acid in 80% Acetonitrile) to 35% for 40 min and followed by an increase to 45% in 5 min and a second increase to 99% in 0.5 min and then kept constant for equilibration for 14.5 min. A full MS was acquired in profile mode using a Q Exactive HF-X Hybrid Quadropole-

Orbitrap mass spectrometer, operating in the scan range of 375-1400 m/z using 120K resolving power with an AGC of 3x 10^6 and max IT of 60ms followed by data independent analysis using 8 Th windows (39 loop counts) with 15K resolving power with an AGC of $3x10^5$ and max IT of 22 ms and a normalized collision energy (NCE) of 26. Each biological replica was analyzed in three technical replicas on the system.

Data Analysis. Orbitrap raw data were analyzed in DIA-NN 1.9.2 (Data-Independent Acquisition by Neural Networks) (7) through searching against the reference *C. elegans and E. coli* Uniprot databases supplemented with the common contaminant database for *C. elegans* and against the reviewed *Mus musculus* Uniprot database for mice. The search was performed using the library-free mode of the software, allowing up to two tryptic missed cleavages. A spectral library was created by the DIA runs and used to reanalyze them. DIA-NN default settings have been used with oxidation of methionine residues and acetylation of the protein N-termini set as variable modifications and carbamidomethylation of cysteine residues as fixed modification. N-terminal methionine excision was also enabled. The match between runs (MBR) feature was used for all analyses and the output (precursor) was filtered at 1% FDR and finally the protein inference was performed on the level of genes using only proteotypic (unique) peptides. The cross-run normalization feature was also activated. Data analyzed to produce the results presented are included in the supplementary data. The generated results were processed statistically and visualized in the Perseus software (1.6.15.0) (8). The mass spectrometry proteomics data have been deposited to the ProteomeXchange Consortium via the PRIDE partner repository with the dataset identifiers PXD073360 (*C. elegans dataset*) (9) and PXD073466 (*M. musculus dataset*) (10). The "unique genes" search engine intensity values/results were log2 transformed and filtered for potential contaminants such as the *E. coli* proteins. The biological and technical replicates were grouped into groups and the groups were filtered for valid values present in 70% in at least one group. Remaining empty values were imputed based on normal distribution. The groups were compared using a Student t-test using permutation-based FDR calculation and the results were visualized in the form of heat maps. Enrichment analyses of statistically significant deregulated proteins were performed using online tools such as GeneCodis 4 and all the graphs were prepared using the GraphPad Prism 9 (GraphPad Software, San Diego, CA, USA).

Statistical analysis. Data were analyzed by using GraphPad Prism 8 and 9 (GraphPad Software, San Diego, CA, USA) software, and results expressed either as the mean ± SEM of the number of experiments performed in replicates of the number of animals indicated in the figure legends. Statistical significance was assessed using the unpaired Student's t-test, one-way or two-way ANOVA with Tukey's multiple comparisons test. Sample normality was tested using Shapiro–Wilk test. Statistical significance was accepted at $P < 0.05$.

Figures

A

```
CLUSTAL O(1.2.4) multiple sequence alignment


PTL-1,                      ------------------------------------------------------------      0
sp|P10637.3|TAU_MOUSE       MADPRQEFDTMEDHAGDYTLLQDQEGDMDHGLKESPPQPPADDGAEEPGSETSDAKSTPT      60

PTL-1,                      ------------------------------------------------------------      0
sp|P10637.3|TAU_MOUSE       AEDVTAPLVDERAPDKQAAAQPHTEIPEGITAEEAGIGDTPNQEDQAAGHVTQGRREGQA      120

PTL-1,                      ------------------------------------------------------------      0
sp|P10637.3|TAU_MOUSE       PDLGTSDWTRQQVSSMSGAPLLPQGLREATCQPSGTRPEDIEKSHPASELLRRGPPQKEG      180

PTL-1,                      -----------------------------------MSTPQSE-------PGSEPGSPGEN      18
sp|P10637.3|TAU_MOUSE       WGQDRLGSEEEVDEDLTVDESSQDSPPSQASLTPGRAAPQAGSGSVCGETASVPGLPTEG      240
                                                            ::**:          .* ** * *.

PTL-1,                      ENSMETQYSQGSFPEVAVEPEPEPEPEPEPEPEPEPEPEPEPEPKPKPEVEPVPQPEPEP      78
sp|P10637.3|TAU_MOUSE       SVPLPADF----FSKVSAETQASQPEGPGTGPMEE-----------------GHEAAPEF      279
                            .  : :::    * :*:.* : .    *   *  *                 :  **

PTL-1,                      EYQPEPEPEPEVEPEPEP---EPVPEPEPEPEPEPEPVVE-KEEEVVVESPPREQEPEKS      134
sp|P10637.3|TAU_MOUSE       TFHVEIKASTPKEQDLEGATVVGVPGEEQKAQT-QGPSVGKGTKEASLQEPPGKQ-PA--      335
                             :: * : .   * : *     **  * : :    * *    :*. ::.** :* *

PTL-1,                      GKSKPSSPIPDAPTME-----------------------------------------D--      151
sp|P10637.3|TAU_MOUSE       -AGLPGRPVSRVPQLKARVASKDRTGNDEKKAKTSTPSCAKAPSHRPCLSPTRPTLGSSD      394
                              . *. *:  .* ::                                         .

PTL-1,                      ---------------IAPRELESLNFSE---------TSGTSDQQADRIMQNNENERV--      185
sp|P10637.3|TAU_MOUSE       PLIKPSSPAVSPEPATSPKHVSSVTPRNGSPGTKQMKLKGADGKTGAKIATPRGAASPAQ      454
                                            :*:.:.*:.  :          .*:..: . :*   .

PTL-1,                      --EEKKQMSPTPSQPQHKTPQRSGIRPPTA------ILRQP-----KPIPASLPRPATAT      232
sp|P10637.3|TAU_MOUSE       KGTSNATRIPAKTTPSPKTPPGSGEPPKSGERSGYSSPGSPGTPGSRSRTPSLPTPPTRE      514
                              .:    *: : *. ***  **  * :.         .*      :    *** * *

PTL-1,                      PSSQRAISTPRQTASTAPSPRPISKMSRERSDVQKSTSTRSIDNVGRMTPKVNAKFVNVK      292
sp|P10637.3|TAU_MOUSE       PKKVAVVRTPPKSP--------------------SASK----SRLQTAP-----------      539
                            *..  .: ** ::                     *:*.    .  : :*

PTL-1,                      SKVGSVTNHKAGGGNVEIFSEKRLYNAQSKVGSLKNATHVAGGGNVQIENRKLDFSAASP      352
sp|P10637.3|TAU_MOUSE       ------------------VPMPDLKNVRSKIGSTENLKHQPGGGKVQIINKKLDLSNVQS      581
                                              .    * *.:**:** :* .*  ***:*** *:***:* ..

PTL-1,                      KVGSKTNYQPA--KSDVKIVSEKL-TWQAKSKVGSMDNAAHKPAGGNVQILSQKLNWKA-      408
sp|P10637.3|TAU_MOUSE       KCGSKDNIKHVPGGGSVQIVYKPVDLSKVTSKCGSLGNIHHKPGGGQVEVKSEKLDFKDR      641
                            * *** * : .   ..*:** : :   :..** **:.*  ***.**:*:: *:**::*

PTL-1,                      -ESKVGSKDNMNHRPGGGNVQIFDEKIRYVSTDSSRNHSTLDISSL--------------      453
sp|P10637.3|TAU_MOUSE       VQSKIGSLDNITHVPGGGNKKIETHKLTFRENAKAKTDHGAEIVYKSPVVSGDTSPRHLS      701
                             :**:** **:.* ***** :*  .*: : .. .::..   :*

PTL-1,                      -------------------------------- 453
sp|P10637.3|TAU_MOUSE       NVSSTGSIDMVDSPQLATLADEVSASLAKQGL 733
```

B

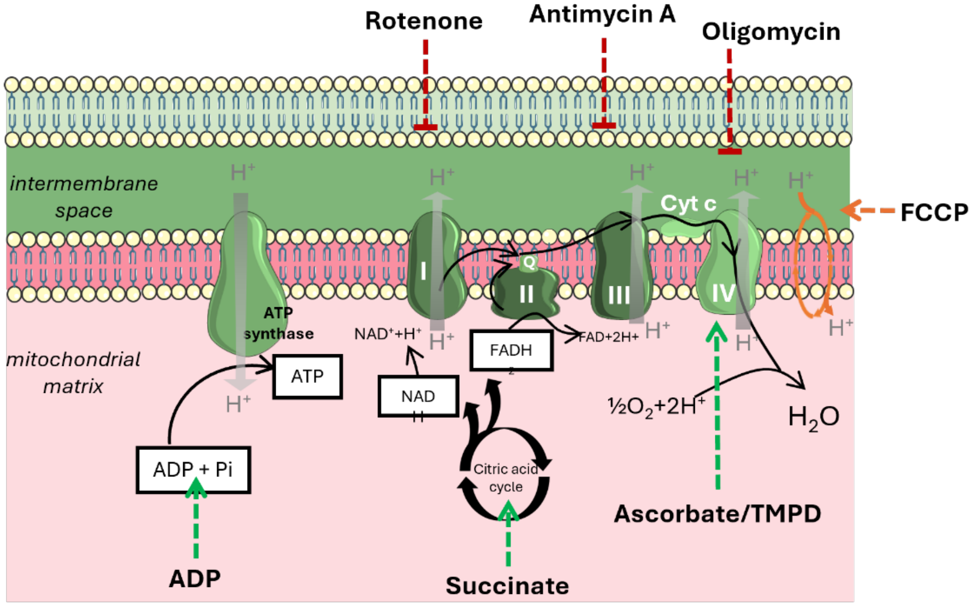

Fig. S1. Sequence alignment of Tau/PTL-1 and schematic of mitochondrial respiratory chain assays. (*A*) Sequence alignment of mouse Tau and *C. elegans* PTL-1 generated using Clustal software. The amino acid sequence of worm PTL-1 (top) and mouse Tau (bottom) is shown. The tubulin-binding domains of mouse Tau are highlighted in pink, while the corresponding regions in PTL-1 are indicated in green and yellow. The deletion in the *ptl-1(ok621)* mutant is indicated in black. (*B*) Schematic representation of the mitochondrial electron transport chain (ETC) and the sites of action of key substrates and inhibitors used in Seahorse-based bioenergetic analyses. The diagram illustrates the inner mitochondrial membrane, including ETC complexes I-IV, ATP synthase, and the flow of electrons and protons (H^+). Substrate entry points (e.g., ADP, succinate) are indicated by green arrows, while the sites of action of rotenone (complex I), antimycin A (complex III), oligomycin (ATP synthase), and FCCP (uncoupler) are shown with dashed lines. Electron transfer from NADH and $FADH_2$ to oxygen, leading to water formation and ATP synthesis, is depicted.

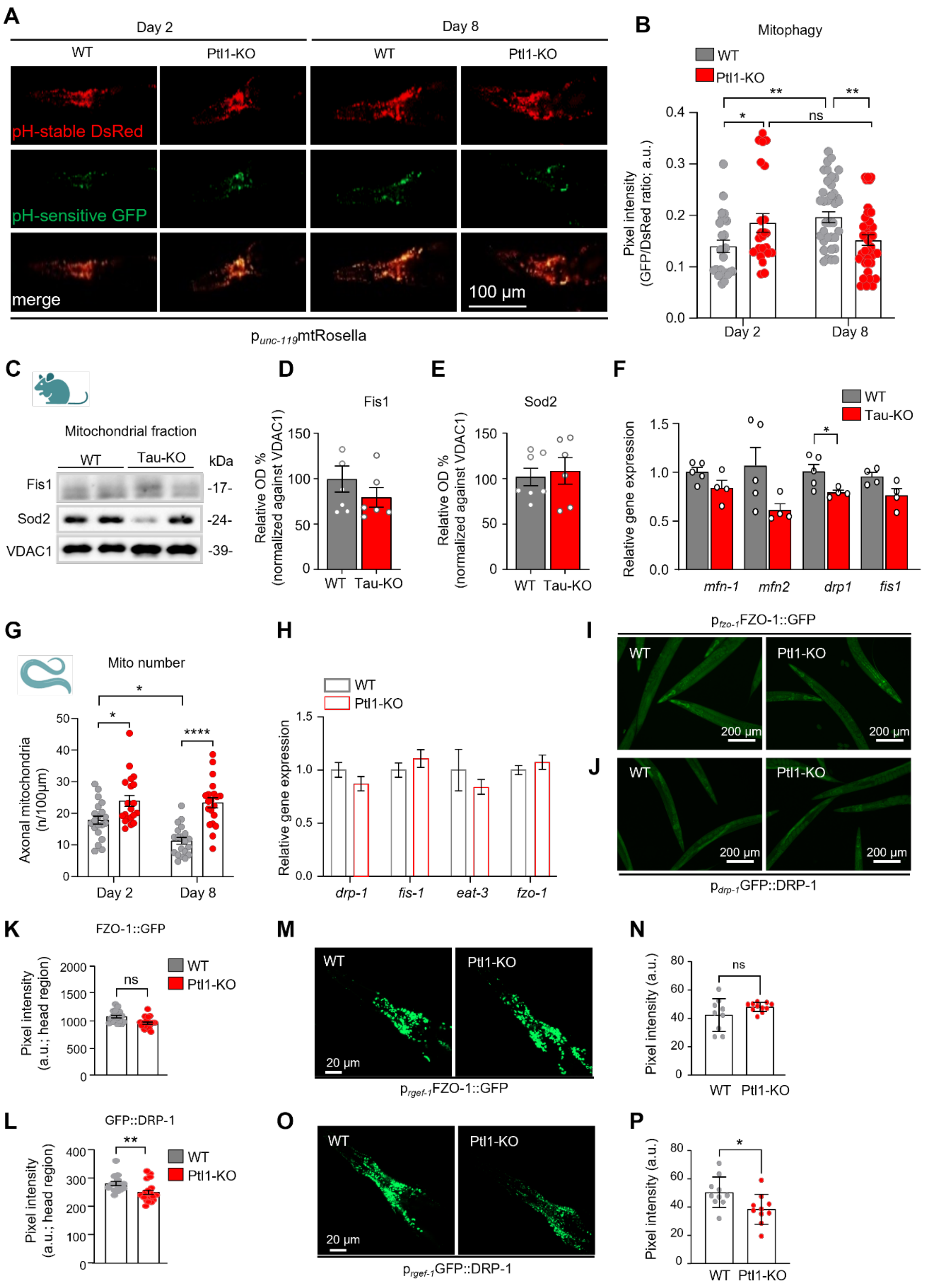

Fig. S2. Expression and mitochondrial phenotypes in Tau/PTL-1 deficient models. (*A, B*) Representative fluorescence images (*A*) and quantification of mitophagy based on the GFP/DsRed

fluorescence ratio (*B*) in the head region of WT and Ptl1-KO worms (scale bar, 100 μm). WT animals exhibited an age-dependent increase in the GFP/DsRed ratio, indicative of reduced mitophagy, whereas Ptl1-KO worms maintained lower ratios during aging, consistent with sustained mitophagy. At day 8, Ptl1-KO worms showed increased mitophagy compared to WT. Data are presented as mean± SEM [n=3 independent experiments; N=20-30 nematodes/group/replicate; ns *P*>0.05 , **P*<0.05, ***P*<0.01, two-way ANOVA with Tukey's multiple comparison test]. (*C*-*E*) Representative immunoblots (*C*) and quantification of mitochondrial Fis1 (*D*) and Sod2 (*E*) protein levels in WT and Tau-KO mouse brain, showing no significant differences between genotypes. Data are presented as mean± SEM [n=6-7 mice/group; ns *P*>0.05, unpaired t-test]. (*F*) Gene expression analysis of mitochondrial fusion (Mfn1, Mfn2) and fission (Drp1, Fis1) regulators in WT and Tau-KO mouse brain revealed no significant differences between groups. Data are presented as mean± SEM [n=4-5 mice/group; ns *P*>0.05, * *P*<0.05, unpaired t-test]. (*G*) Quantification of axonal mitochondria in GABAergic neurons of WT and Ptl1-KO worms showed increased mitochondrial content in Ptl1-KO animals at both day 2 and day 8. Data are presented as mean± SEM, with each data point representing a single nematode's axon [n=3 independent experiments; N=10-15 nematodes/ group/replicate, **P*<0.05, *****P*<0.0001, two-way ANOVA with Tukey's multiple comparison test]. (*H*) Gene expression analysis of mitochondrial fusion (*fzo-1, eat-3*) and fission (*drp-1, fis-1*) genes in WT and Ptl1-KO worms showed no significant differences. Data are presented as mean± SEM [n=3 independent experiments; N=100-200 nematodes/group/replicate; ns *P*>0.05, unpaired t-test]. (*I-L*) Representative images (*I, J*) and quantification of total FZO-1 (*K*) and DRP-1 (*L*) levels in the head region of transgenic worms expressing endogenous FZO-1::GFP or GFP::DRP-1 in WT and Ptl1-KO backgrounds. No significant differences were observed in FZO-1 levels, whereas DRP-1 levels were reduced in Ptl1-KO animals (scale bar, 200 μm). Data are presented as mean± SEM [n=2 independent experiments; N=15-20 nematodes/group/replicate; ns *P*>0.05, ***P*<0.01, unpaired t-test]. (*M-P*) Representative images (*M, O*) and quantification of neuronal FZO-1 (*N*) and DRP-1 (*P*) levels in transgenic worms expressing FZO-1::GFP or GFP::DRP-1 under the pan-neuronal promoter *rgef-1*. FZO-1 levels were unchanged, whereas DRP-1 levels were reduced in Ptl1-KO animals (scale bar, 20 μm). Data are presented as mean± SEM [n=2-3 independent experiments; N=10-15 nematodes/group/replicate; ns *P*>0.05, **P*<0.05, unpaired t-test].

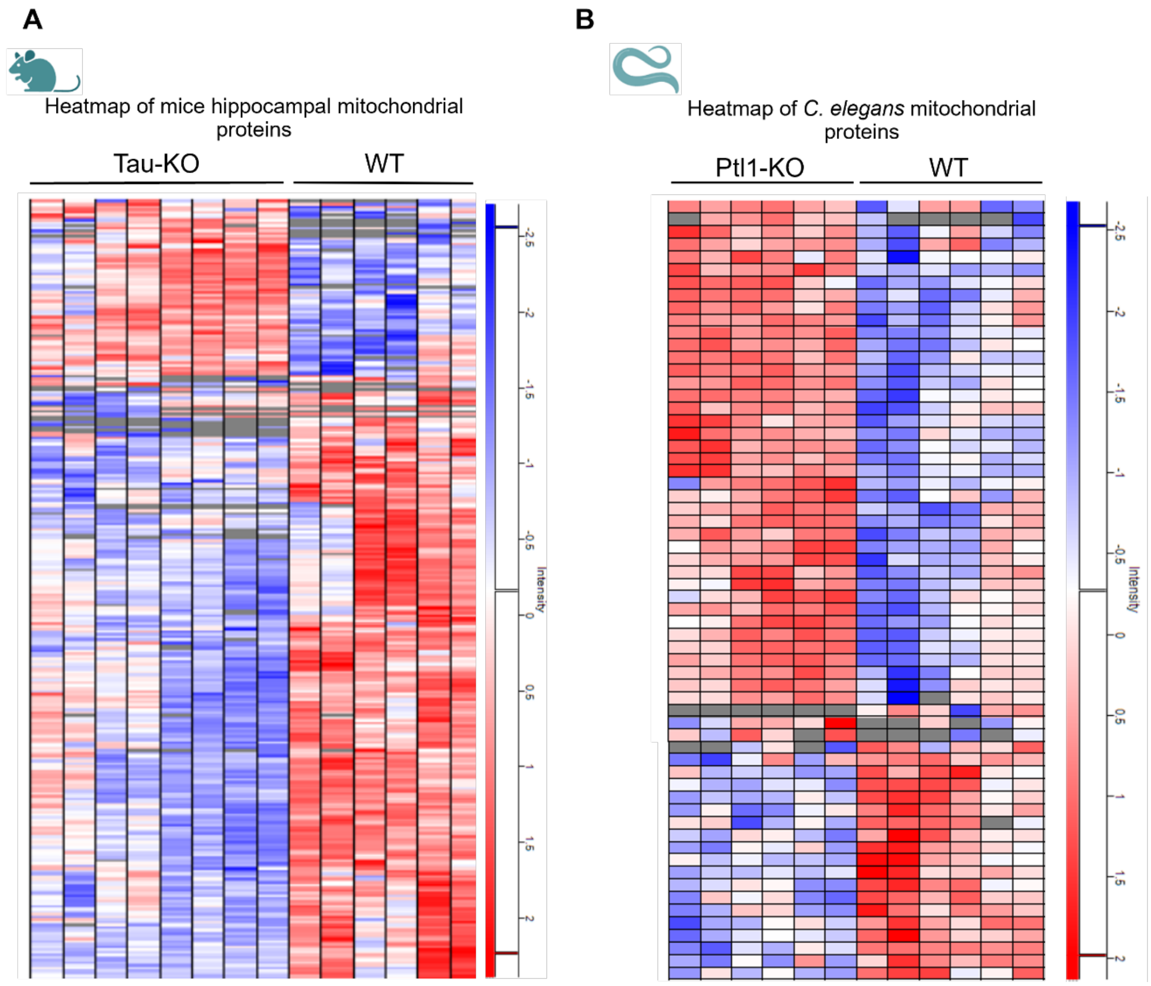

Fig. S3. Proteomic analysis reveals differential regulation of mitochondrial proteins in Tau/PTL-1 deficient models. (*A, B*) Heatmaps of mitochondrial protein expression in mice (*A*) and worms (*B*), showing distinct clustering between Tau/PTL-1–deficient and WT samples. Protein expression levels are represented by a color gradient ranging from low (blue) to high (red).

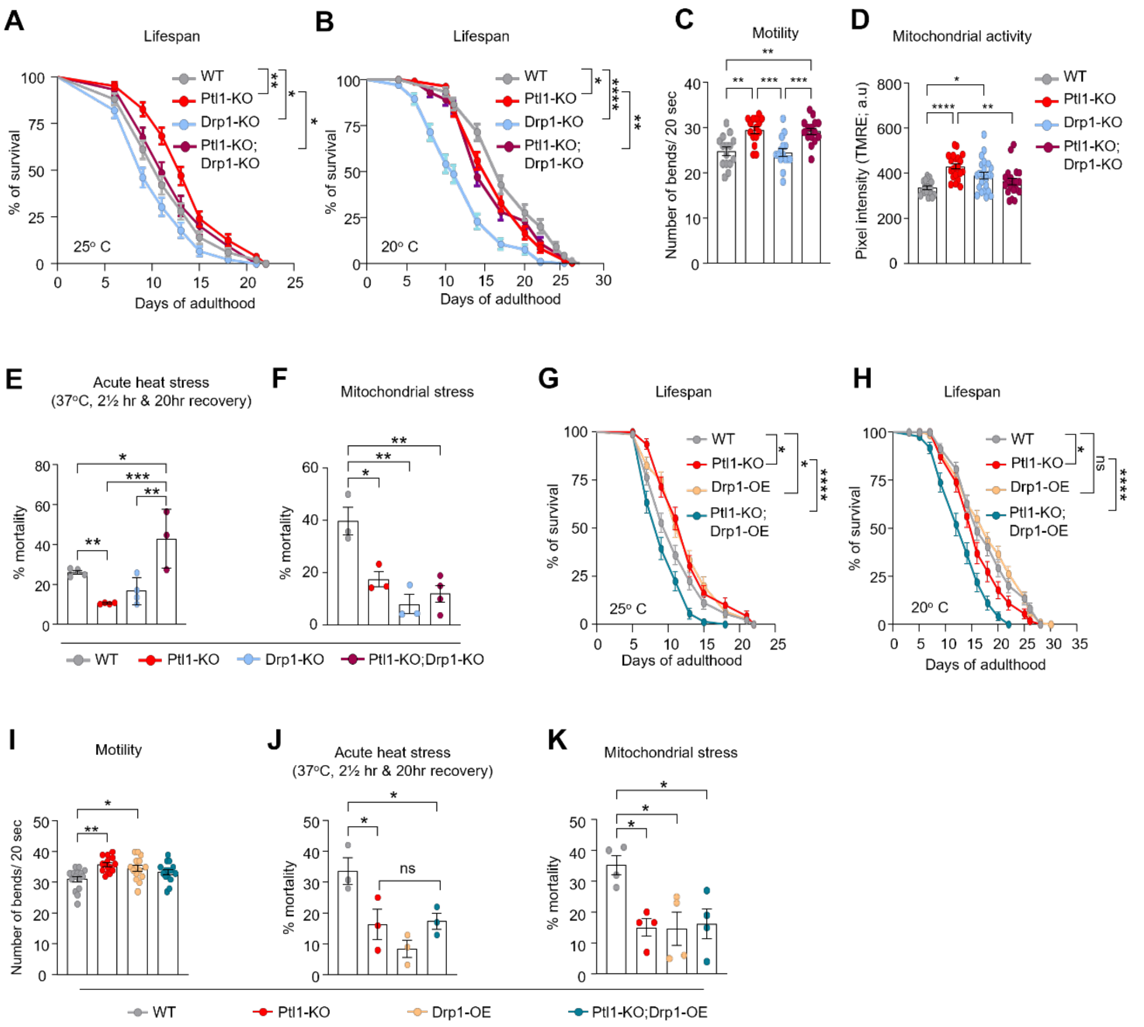

Fig. S4. Modulation of DRP-1 has limited effects on PTL-1/Tau-dependent mitochondrial and physiological phenotypes. (*A, B*) Survival analysis showing that Ptl1-KO/Drp1-KO double-mutant worms display lifespan comparable to Ptl1-KO animals at both 25°C (*A*) and 20°C (*B*), whereas Drp1-KO alone results in reduced lifespan compared to WT. Survival curves were analyzed by Kaplan-Meier statistical analysis followed by Log-rank/Mantel-Cox test (see *SI Appendix*, Table S3) [n=3 independent experiments; N=70-120 nematodes/group/replicate; **P*<0.05, ***P*<0.01, ****P*<0.001, *****P*<0.0001]. (*C*) Ptl1-KO worms exhibited increased motility compared to WT, which was not significantly altered in Ptl1-KO/Drp1-KO animals. Data are presented as mean± SEM [n=3 independent experiments; N=10-15 nematodes/group/replicate; ***P*<0.01, ****P*<0.001, one-way ANOVA with Tukey's multiple comparison test]. (*D*) Mitochondrial membrane potential, assessed by TMRE fluorescence, was elevated in Ptl1-KO worms and reduced to levels comparable to WT in Ptl1-KO/Drp1-KO animals. Data are presented as mean± SEM [n=3 independent experiments; N=15-25 nematodes/group/replicate; **P*<0.05, ***P*<0.01, *****P*<0.0001, one-way ANOVA with Tukey's multiple comparison test]. (*E*) Under acute heat stress, Ptl1-KO/Drp1-KO animals exhibited

increased sensitivity compared to Ptl1-KO worms and were similar to WT. (*F*) Ptl1-KO worms showed reduced mortality under mitochondrial stress induced by antimycin A compared to WT, and this phenotype was not significantly altered in Ptl1-KO/Drp1-KO animals. Data are presented as mean± SEM [n=3-4 independent experiments; N=25-30 nematodes/group/replicate; **P*<0.05, ***P*<0.01, ****P*<0.001, one-way ANOVA with Tukey's multiple comparison test]. (*G, H*) Overexpression of DRP-1 (Drp1-OE) in the Ptl1-KO background reduced lifespan compared to Ptl1-KO worms at both 25°C (*G*) and 20°C (*H*), while Drp1-OE alone increased lifespan relative to WT. Survival curves were analyzed by Kaplan-Meier statistical analysis followed by Log-rank/Mantel-Cox test (see *SI Appendix*, Table S3) [n=3 independent experiments; N=90-150 nematodes/group/replicate; ns *P*>0.05, **P*<0.05, *****P*<0.0001]. (*I-K*) Motility (*I*), resistance to acute heat stress (*J*), and mitochondrial stress (*K*) in Ptl1-KO worms were not significantly affected by DRP-1 overexpression. For the motility assay (*I*) data are presented as mean± SEM [n=3 independent experiments; N=10-15 nematodes/group/replicate; **P*<0.05, ***P*<0.01, one-way ANOVA with Tukey's multiple comparison test]. For the mortality assessment upon acute heat stress and mitochondrial stress (*J, K*) data are presented as mean± SEM [n=3-4 independent experiments; N=25-30 nematodes/group/replicate; ns *P*>0.05, **P*<0.05, one-way ANOVA with Tukey's multiple comparison test].

Tables

Table S1. *C. elegans* strains used in this study

Strain	Genotype	Available from
	N2: wild-type Bristol isolate	CGC
RB809	*ptl-1(ok621)*	CGC; OMRF Knockout Group
EG6531	*oxIs608*[p_{unc-47}mCherry]; *oxEx1182*[p_{unc-47} TOMM-20::GFP]	E.M. Jorgensen lab; Rawnson et al., 2014
KPA129	*ptl-1(ok621);* [p_{unc-47}mCherry]; *oxEx1182*[p_{unc-47}TOMM-20::GFP]	Palikaras lab
IR1864	N2; *Ex001*[$p_{unc-119}$TOMM-20::Rosella; pRF4]	Tavernarakis Lab
KPA358	*ptl-1(ok621); Ex001*[$p_{unc-119}$TOMM-20::Rosella + *rol-6(su1006)*]	Palikaras Lab
CU5991	*fzo-1(tm1133)*	CGC
KPA359	*ptl-1(ok621); fzo-1(tm1133)*	Palikaras Lab
SJZ216	*foxSi44* [p_{rgef-1}TOMM-20::mKate2::HA::*tbb-2* 3'UTR] I.	CGC
KPA517	*ptl-1(ok621);* [p_{rgef-1}TOMM-20 ::mKate2::HA::*tbb-2* 3'UTR]	Palikaras Lab
CU6372	*drp-1(tm1108) IV*	CGC
JVR589	*sybIs3776* [p_{rpl-28}FZO-1::*unc-54* 3′UTR + p_{myo-2} mCherry]	Jeremy M. Van Raamsdonk Lab
KPA521	*ptl-1(ok621)*; [p_{rpl-28}FZO-1::*unc-54* 3′UTR + p_{myo-2} mCherry]	Palikaras Lab
KPA565	*ptl-1(ok621);* [p_{rpl-28}FZO-1::*unc-54 3'UTR* + p_{myo-2}mCherry]; *oxIs608* [p_{unc-47}mCherry]*; oxEx1182* [p_{unc-47}TOMM-20::GFP]	Palikaras Lab
KPA566	[p_{rpl-28}FZO-1::*unc-54* 3′UTR + p_{myo-2}mCherry]; *oxIs608* [p_{unc-47}mCherry]*; oxEx1182* [p_{unc-47} TOMM-20::GFP]	Palikaras Lab
KPA520	*ptl-1(ok621)*; *fzo-1(tm1133)*; *oxEx1182* [p_{unc-47} TOMM-20::GFP]	Palikaras Lab
KPA564	*fzo-1(tm1133)*; *oxIs608* [p_{unc-47}mCherry]; *oxEx1182* [p_{unc-47}TOMM-20::GFP]	Palikaras Lab
KPA522	*ptl-1(ok621)*; *drp-1(tm1108)*	Palikaras Lab

EU2917	*drp-1*(*or1941*[GFP::DRP-1])IV	CGC
KPA519	*ptl-1(ok621); drp-1*(*or1941*[GFP::DRP-1])	Palikaras Lab
SHX324	*fzo-1(zju136*[FZO-1::GFP]*)* II	CGC
KPA523	*ptl-1(ok621); fzo-1(zju136*[FZO-1::GFP]*)*	Palikaras Lab
KPA559	N2; *Ex001*[p_{rgef-1}GFP::DRP-1 + *rol-6(su1006)*]	Palikaras Lab
KPA556	*N2; Ex001*[p_{rgef-1} FZO-1::GFP + *rol-6(su1006)*]	Palikaras Lab
KPA563	*ptl-1(ok621); Ex001*p_{rgef-1}FZO-1::GFP + *rol-6 (su1006)*]	Palikaras Lab
KPA561	*ptl-1(ok621); Ex001*[p_{rgef-1}GFP::DRP-1+ *rol-6 (su1006)*]	Palikaras Lab

Table S2. Primers used in this study

Name of gene	Sequence (5´-3´)
***C. elegans* primers**	
*drp-1*_RT_FW	TCCACAGATTGTAGTCGTCGGATCAC
*drp-1*_RT_RV	CGCACGGCATCGAAGTCTGTG
*fzo-1*_RT_FW	ATACAACATGTTGCTTTCTGCAGGTTG
*fzo-1*_RT_RV	GATCGACACCAGGGCTATCAAGTATG
*eat-3*_RT_FW	CAATTAGGTTGAAACGGCTTAAGAGATC
*eat-3*_RT_RV	TCACTGGAGCTCTTGTCATCATTTCTC
*fis-1*_RT_FW	CAAGGAAGGAATTGAAATTCTTGAAGATGTAG
*fis-1*_RT_RV	AGTCCCAGAAGACCTTCACGTTTC
*ama-1*_RT_FW	GCTATGGTGCCGAGACAAC
ama-1_RT_RV	CCAGGAATGATAAGCGAGAAGAC
**Mouse primers**	
mfn1_FW	TATGAGAGGCTGACGTGGAC
mfn1_RV	GTTTGCACTGGTGAAGCTCA
mfn2_FW	CTGTGCCAGCAAGTTGACAT
mfn2_RV	TGGCTCTGCTCTGAAGTGAA
drp1_FW	ATGCCAGCAAGTCCACAGAA
drp1_RV	TGTTCTCGGGCAGACAGTTT
fis1_FW	GCCCCTGCTACTGGACCAT
fis1_RV	CCCTGAAAGCCTCACACTAAGG

Table S3. Statistical analysis of Survival assays.

All survival assays were performed at least three independent times on NGM plates seeded with OP50 at 20°C, unless otherwise indicated. For each lifespan assay corresponding to the figures, median and maximum lifespan (corresponding to 50% mortality and 10% survival, respectively), as well as the total number of animals and censored animals, are reported. Statistical significance was assessed using the log-rank (Mantel–Cox) test, and the corresponding p-values are shown.

Corresponding Fig.	Strain	Treatment	Median	Max	N (T/C)	p-value (vs ctrl)
Fig. 3G	WT	-	16	23	117/18	-
	Ptl1-KO	-	14	21	87/3	0.0088 (**) vs WT
	WT	10mM NAC	16	21	83/38	0.5122 (ns) vs WT
	Ptl1-KO	10mM NAC	14	21	82/23	0.0687 (ns) vs Ptl1-KO, 0.9139 (ns) vs WT-NAC
Fig. 3H	WT	25°C	12	21	106/0	-
	Ptl1-KO	25°C	14	21	107/4	0.0312 (*) vs WT
	WT	10mM NAC/ 25°C	12	18	93/19	0.9151(ns) vs WT
	Ptl1-KO	10mM NAC/ 25°C	12	18	89/12	0.0247 (*) vs Ptl1-KO, 0.9376 (ns) vs WT-NAC
Fig. 4C	WT	25°C	11	18	150/4	-
	Ptl1-KO	25°C	12	18	150/6	0.2025 (ns) vs WT
	Fzo1-KO	25°C	12	21	150/9	0.0318 (*) vs WT
	Ptl1-KO; Fzo1-KO	25°C	10	18	150/11	<0.0001 (****) vs Ptl1-KO, <0.0001 (****) vs Fzo1-KO, 0.0035 (**) vs WT
Fig. 4D	WT	-	17	24	109/4	-
	Ptl1-KO	-	17	22	117/8	0.0413 (*) vs WT
	Fzo1-KO	-	14	22	102/2	<0.0001 (****) vs WT
	Ptl1-KO; Fzo1-KO	-	10	17	73/0	<0.0001 (****) vs Ptl1-KO, <0.0001 (****) vs Fzo1-KO, <0.0001 (****) vs WT
Fig. 5A	WT	25°C	11	18	90/12	-
	Ptl1-KO	25°C	13	18	80/8	0,0185 (*) vs WT
	Fzo1-OE	25°C	13	21	76/6	<0.0001 (****) vs WT
	Ptl1-KO; Fzo1-OE	25°C	15	21	75/12	0.0094 (**) vs Ptl1-KO, 0.6266 (ns) vs Fzo1-OE, <0.0001 (****) vs WT
Fig. 5B	WT	-	16	26	109/25	-
	Ptl1-KO	-	16	22	73/19	0.0299 (*) vs WT
	Fzo1-OE	-	22	26	106/39	0.0004 (***) vs WT
	Ptl1-KO; Fzo1-OE	-	20	27	110/32	<0.0001 (****) vs Ptl1-KO, 0.5378 (ns) vs Fzo1-OE, 0.0035(**) vs WT

Fig. S4A	WT	25°C	11	18	100/0	-
	Ptl1-KO	25°C	13	21	104/0	0,0012 (**) vs WT
	Drp1-KO	25°C	9	17	89/0	0.0225 (*) vs WT
	Ptl1-KO; Drp1-KO	25°C	11	21	74/0	0.0331 (*) vs Ptl1-KO, 0.0017 (**) vs Drp1-KO, 0.3511 (ns) vs WT
Fig. S4B	WT	-	17	25	109/4	-
	Ptl1-KO	-	17	21	117/8	0.0413 (*) vs WT
	Drp1-KO	-	11	20	105/0	<0.0001 (****) vs WT
	Ptl1-KO; Drp1-KO	-	14	25	108/8	0.9193 (ns) vs Ptl1-KO, <0.0001 (****) vs Drp1-KO, 0.0726 (ns) vs WT
Fig. S4G	WT	25°C	11	18	107/12	-
	Ptl1-KO	25°C	13	19	83/12	0.0185 (*) vs WT
	Drp1-OE	25°C	13	19	90/12	0.0329(*) vs WT
	Ptl1-KO; Drp1-OE	25°C	9	16	80/8	<0.0001 (****) vs Ptl1-KO, <0.0001 (****) vs Drp1-OE, 0.0024 (**) vs WT
Fig. S4H	WT	-	16	26	134/25	-
	Ptl1-KO	-	16	24	92/19	0.0299 (*) vs WT
	Drp1-OE	-	18	26	145/9	0.6715 (ns) vs WT
	Ptl1-KO; Drp1-OE	-	14	20	86/11	0.0003 (***) vs Ptl1-KO, <0.0001 (****) vs Drp1-OE, <0.0001 (****) vs WT

Table S4. Top 60 most significantly altered proteins in the proteomic analysis of WT and Ptl1-KO *C. elegans*.

	WT						Ptl1-KO						
**Genes**	**37_1**	**37_2**	**38_1**	**38_2**	**39_1**	**39_2**	**40_1**	**40_2**	**41_1**	**41_2**	**42_1**	**42_2**	**N: -Log Student's T-test p-value Control_Ptl-1_KO**
gfi-1	26.7687	26.963	26.2335	26.2389	26.471	26.7862	17.4563	17.5254	17.1775	17.7705	17.506	17.8992	13.0559
bcf-1	18.0396	19.558	18.4739	18.5065	17.9402	18.9543	26.6661	26.4959	26.6212	26.7434	26.5109	26.9238	10.6481
CELE_Y39B6A.1	24.3046	23.9014	24.3573	23.9117	23.7407	23.7079	26.9517	26.871	26.9142	26.8612	26.8382	27.0737	9.54679
col-39	19.682	19.9421	20.5322	20.6622	20.4225	19.952	24.4316	24.7916	24.7899	25.0651	25.0275	25.2348	9.40486
bli-1	18.338	17.876	18.5408	17.5539	18.1968	17.6724	22.7996	22.7526	23.5415	23.5616	23.6159	23.5934	9.20739
fln-2	23.507	23.5316	23.3171	23.4091	23.4146	23.3443	22.267	22.1772	22.3105	22.2826	22.4753	22.2896	8.91217
Y51H7C.13	22.4104	22.3395	21.6053	21.9564	22.0065	22.1792	25.3731	25.4039	26.0566	26.0742	25.5504	25.5642	8.74854
grd-6	17.27	17.5682	17.7379	17.9761	17.9826	18.4707	22.3833	22.3257	23.0187	22.853	22.4414	21.8594	8.57102
scb-1	18.2695	17.6882	18.6291	17.944	17.8077	17.7792	21.5186	21.6254	22.4406	21.5523	21.8239	21.9982	8.33996
CELE_T04A11.1	21.6286	21.5706	21.5548	21.7515	21.6538	21.6653	22.8335	22.8336	22.8578	23.0153	22.6332	22.6443	8.16241
grl-16	17.8259	17.3988	18.0724	18.2156	16.6601	19.3131	24.5796	25.1286	26.0355	26.0534	25.0532	25.3693	8.03954
asp-12	23.6631	23.637	23.3868	23.5236	23.6336	23.5914	22.5522	22.4704	22.5361	22.4106	22.7168	22.401	7.88316
gst-5	18.6817	17.3554	19.5463	19.1524	18.4719	17.8517	24.0133	24.1128	23.8022	23.7317	24.0687	24.2147	7.76346
ifb-2	26.4444	26.4948	26.3214	26.3391	26.4262	26.3651	26.8711	26.8703	26.9765	26.9718	26.958	26.953	7.64267
pes-8	21.8176	21.9133	21.7874	21.8177	21.8859	21.7824	22.3744	22.4623	22.635	22.5744	22.5855	22.5723	7.60279
bli-2	19.1501	19.361	19.7912	20.1809	20.1105	20.0246	22.5887	22.672	23.0217	23.0286	23.2524	23.3165	7.50691
B0205.13	21.9924	22.0504	21.7119	22.114	22.0892	22.1226	23.1308	23.282	23.108	23.1529	23.0732	23.3699	7.42108
ZK550.6	24.3387	24.2875	24.2922	24.2798	24.2738	24.3188	24.0559	24.0377	24.0136	23.9519	24.0598	23.9873	7.16993
CELE_ZK180.6	17.7472	18.5759	17.2137	17.8186	19.3997	18.2016	22.9405	22.5358	23.967	23.841	23.6678	23.5774	7.05969
clec-63	26.1084	26.1034	25.9632	26.1109	26.134	26.0288	26.554	26.6403	26.5002	26.5305	26.5948	26.4767	6.87609
CELE_T21C9.9	18.7345	18.2679	17.2578	18.4014	17.842	18.5093	21.0824	21.2096	22.1	22.1872	21.7711	21.4994	6.5605
haf-6	22.4246	22.3795	21.9279	21.5909	22.2534	22.1319	17.8851	17.7981	17.3744	18.3388	19.5419	18.2406	6.49983
irg-7	25.0575	25.017	24.6958	24.7365	24.83	24.7785	25.6166	25.6389	25.5739	25.5765	25.6616	25.7562	6.41589
flor-1	20.7597	20.7471	20.6958	20.6155	20.7317	20.745	21.6577	21.5843	21.3652	21.3536	21.646	21.3318	6.37318
dpf-3	22.8245	22.8096	22.7998	22.747	22.7271	22.7495	23.0943	23.0209	23.1029	23.0422	23.0015	23.0197	6.32994
ule-5	25.5181	25.4792	25.4531	25.4296	25.6341	25.5065	26.2952	26.1639	26.6261	26.6299	26.5925	26.5241	6.31204

cpr-6	25.6877	25.677	25.8421	25.876	25.7013	25.7763	25.2232	25.3102	25.353	25.3749	25.2752	25.3226	6.15201
dpy-8	17.0803	17.881	17.4258	18.5072	19.1432	18.3932	21.3609	21.4539	21.9214	21.9359	22.472	22.0412	6.10192
CELE_T25C12.3	25.0007	25.0079	24.6876	24.78	24.8085	24.7194	25.5324	25.5772	25.4171	25.4081	25.5413	25.4714	5.94351
CELE_ZK813.3	23.0278	22.8939	23.2398	23.3645	23.5706	23.3529	24.5062	24.2303	24.4248	24.3881	24.3873	24.2644	5.93324
cbl-1	23.3965	23.383	23.3193	23.3277	23.2831	23.318	23.1121	23.0643	23.0824	23.1393	23.0511	23.1393	5.88759
C17F4.7	25.395	25.4859	25.4227	25.429	25.5357	25.4285	26.028	26.0713	25.8762	26.0367	25.8088	25.8776	5.87626
lim-8	21.583	21.6278	21.5224	21.4699	21.6871	21.5809	21.9483	21.9264	21.8904	21.9408	21.9706	21.883	5.87408
col-63	19.037	18.72	16.98	17.994	17.793	17.188	21.9877	22.162	21.4258	21.5507	22.9234	22.6615	5.84572
lptD	21.073	21.1725	21.192	21.2104	21.2317	21.0511	21.6227	21.5534	21.5639	21.5646	21.7271	21.5129	5.80971
CELE_W04B5.3	21.4227	21.4516	21.2581	21.3544	21.3009	21.1405	21.9526	21.9544	21.8205	21.8549	22.0374	21.8156	5.74478
cht-1	22.061	22.151	22.2556	22.4419	22.0466	22.1297	21.324	21.0782	20.812	20.8964	20.594	21.1613	5.70803
col-58	18.3228	18.0389	16.6177	17.6367	18.0399	19.1022	21.0738	21.056	21.7176	21.6102	21.7217	21.9693	5.67458
endu-2	22.2189	22.2002	22.1408	22.1617	22.1661	22.3379	21.8245	21.8607	21.6239	21.7005	21.7672	21.657	5.65719
CELE_Y47G6A.15	24.0892	24.2619	24.3016	23.0581	25.6328	24.3609	27.5284	27.7997	27.6756	27.409	27.38	27.9955	5.63371
ifc-2	25.9738	25.9878	25.9043	25.7932	26.0209	25.9663	26.4356	26.3496	26.7112	26.6249	26.5996	26.5692	5.6193
CELE_T25G3.3	23.7115	23.8241	23.5587	23.7146	23.6113	23.6639	24.0512	24.0227	24.17	24.1078	24.1633	24.141	5.59416
dao-5	24.9047	24.8895	24.7462	24.8263	25.1183	24.9746	25.4024	25.3926	25.4656	25.4239	25.4997	25.3861	5.53651
dppA	23.4767	23.4885	23.2362	23.5391	23.693	23.7308	22.7111	22.7602	22.6432	22.5402	22.7927	22.394	5.52528
CELE_H06I04.3	24.8007	24.8187	24.75	24.731	24.7075	24.7815	24.5605	24.5835	24.5413	24.5049	24.5173	24.5976	5.52311
ftn-2	25.2181	25.2606	25.2434	25.4042	25.0586	25.0169	24.1299	23.9873	24.3741	24.4711	24.14	23.8051	5.41828
CELE_Y11D7A.10	21.7693	21.8293	21.4349	21.7064	21.4134	21.491	20.4661	20.1285	20.7311	20.6925	20.6533	20.629	5.35126
mrpl-36	21.5794	21.6585	21.6381	21.7269	21.5985	21.7769	22.0274	22.3421	22.1616	22.12	22.1906	22.3695	5.24431
slc-25a10	23.3831	23.4315	23.1802	23.0574	23.4499	23.5433	24.0496	24.0615	24.0911	23.8975	24.0237	24.0639	5.21072
sfxn-2	22.538	22.5547	22.1333	22.1568	22.5338	22.2179	23.2484	23.0978	23.1467	23.0293	23.2515	23.0549	5.13198
rps-3	29.6645	29.7319	29.6308	29.6965	29.7481	29.7513	30.0019	30.099	30.059	30.0811	30.071	30.3083	5.11864
col-49	21.8699	21.8745	22.2292	22.4296	22.2467	22.3745	23.3235	23.2386	23.1238	23.2398	23.5375	23.833	5.0856
ppw-2	24.0002	23.8898	23.8215	23.8112	23.8829	24.0278	24.2167	24.1942	24.2184	24.1953	24.2362	24.2052	5.00867
tyr-2	19.1752	18.8536	19.4687	18.6719	18.5535	19.0884	20.3592	20.1321	20.2678	20.4384	20.509	19.9814	5.00635
ZK632.4	23.8125	23.9076	23.8805	23.9112	23.9156	23.9882	24.1691	24.0988	24.1279	24.1338	24.0818	24.0952	4.95885
lact-1	20.7516	20.7605	20.44	20.6954	20.7432	20.6035	20.046	20.0204	19.967	20.0685	20.2172	20.2805	4.90042
haly-1	24.7015	24.7758	24.7111	24.698	24.6842	24.6352	24.5431	24.4311	24.4481	24.4261	24.4059	24.3252	4.89726
tpst-1	22.017	22.053	22.0048	22.0061	21.9832	21.8744	21.6161	21.6126	21.5678	21.3912	21.7206	21.4618	4.87277

elpc-2	21.7765	21.4404	21.5036	21.5452	21.51	21.4336	21.8986	21.9977	22.1354	22.1277	22.1475	21.9991	4.84732
dod-19	25.0973	25.1788	24.6213	24.7904	24.8127	24.6937	25.7243	25.5677	25.5762	25.5302	25.7645	25.7569	4.83165

Table S5. The significantly altered mitochondrial proteins in the proteomic analysis of WT and Ptl1-KO *C. elegans*.

	WT						Ptl1-KO						
**Genes**	**37_1**	**37_2**	**38_1**	**38_2**	**39_1**	**39_2**	**40_1**	**40_2**	**41_1**	**41_2**	**42_1**	**42_2**	**-log(P-value)**
mrpl-36	21.57938	21.65846	21.63807	21.72691	21.59848	21.77689	22.02744	22.34214	22.16158	22.12001	22.19058	22.36947	5.244311215
sfxn-2	22.53802	22.55465	22.13326	22.15684	22.53379	22.2179	23.2484	23.0978	23.14675	23.02928	23.2515	23.05485	5.131983683
mev-1	24.64892	24.62024	24.07635	24.576	25.08891	24.38191	25.71563	25.75682	25.71722	25.85697	25.73225	25.32635	4.533271769
mpc-2	25.09134	25.10297	24.52846	24.8258	24.99749	24.91121	25.60459	25.71271	25.70869	25.58578	25.49404	25.48406	4.498396471
pck-2	28.4844	28.45775	28.50542	28.49707	28.48756	28.47768	28.06073	28.12049	28.18433	28.25382	28.25027	28.32855	4.450420576
tomm-40	25.3925	25.49502	25.30919	25.2384	25.45232	25.52814	25.94553	25.87231	25.74486	25.68745	25.73464	25.75885	4.108597889
K11H3.3	24.11148	24.11486	23.93646	24.11533	24.33459	24.29427	25.13918	25.14408	24.61934	24.66358	24.7134	24.82969	3.999740222
mtch-1	25.39313	25.47752	24.88458	25.07105	25.16044	24.88748	26.01394	25.92266	25.79967	25.74468	25.9321	25.68416	3.969420201
misc-1	24.1756	24.318	23.42719	23.67945	23.80449	23.9363	24.64618	24.64154	24.82919	24.88522	24.78633	24.6593	3.922160657
abtm-1	22.57694	22.43175	22.22124	22.24997	22.74045	22.67907	23.37624	23.32043	23.09325	23.01559	23.09778	22.92934	3.726953883
timm-23	23.90407	23.8879	23.75147	23.807	24.02733	24.18176	24.43789	24.65425	24.3637	24.33764	24.24944	24.28325	3.445733275
F01G4.6	28.83382	28.85072	28.19494	28.18731	28.45666	28.53791	28.91805	29.07357	29.31317	29.24397	29.18868	29.33918	3.218001334
sod-1	26.26093	26.19196	26.52275	26.48307	26.32783	26.49202	26.08003	26.01014	26.05658	26.0358	26.08092	26.21071	3.107489152
mfn-1	21.8183	21.85081	21.72489	21.56056	22.00833	21.87016	22.37688	22.40948	22.00304	22.21766	22.15242	22.13205	3.036293937
trxr-1	21.54947	21.31695	21.29542	21.11592	21.05171	21.01229	21.69571	21.74256	21.70832	21.71688	21.56106	21.48761	3.017856685
dbt-1	25.73742	25.7506	25.85589	25.67221	25.54406	25.64783	25.31857	25.14586	25.48782	25.34185	25.39266	25.54187	3.015080587
immt-1	23.76004	23.81291	23.25982	23.37523	23.71277	23.74351	24.12658	24.11219	24.06937	24.07559	23.95448	23.98854	2.933448406
cyc-2.1	28.37098	28.27753	28.72711	28.71229	28.69522	28.73421	28.13172	28.28214	28.2402	28.09692	28.04266	28.25845	2.887532746
T09A5.5	23.1286	22.95947	23.45457	23.21897	23.13455	23.28303	22.93062	22.63367	22.59154	22.52025	22.95381	22.86042	2.84716159
spg-7	24.87665	24.80795	24.42536	24.47519	24.80693	24.64337	25.01765	24.97507	25.10348	24.92414	25.14107	25.06765	2.839376371
oxa-1	23.21275	23.1911	22.5393	22.57837	23.07369	22.94063	23.39523	23.24491	23.56295	23.68781	23.60438	23.73633	2.800878433
bcat-1	25.90207	25.84945	26.12593	25.9806	25.84418	25.91971	25.68724	25.60535	25.79067	25.75846	25.72503	25.76122	2.761854932
dnj-9	22.7221	22.74198	22.20613	22.1885	22.51088	22.55791	22.77081	22.78197	23.08838	23.07893	23.077	23.05014	2.703159574
R53.4	27.45385	27.32905	27.07893	27.00012	27.00328	26.96167	27.3389	27.4408	27.62544	27.57391	27.59236	27.61007	2.673966873
hpo-19	26.00126	25.93044	25.79686	25.69998	25.89475	25.76726	26.18367	26.2245	26.22444	26.20581	25.9224	26.02812	2.664056961
nuo-4	26.67147	26.74304	26.92215	26.98542	26.69616	26.6722	26.55412	26.49583	26.59048	26.53555	26.58959	26.53786	2.591992645
fzo-1	23.03506	23.16041	22.89178	22.84089	22.87894	22.90623	23.19119	23.03723	23.22427	23.15788	23.32955	23.32844	2.501443902
Y71F9B.2	22.22194	22.07495	21.94496	21.7601	21.98558	21.82626	22.20723	22.24961	22.28779	22.20148	22.21474	22.24949	2.471724648
F25B5.6	23.17889	23.25803	23.08023	22.77182	23.01421	22.99611	23.29754	23.22501	23.33559	23.32876	23.47201	23.49549	2.468855675

B0334.5	20.3824	19.92294	20.48693	20.33118	19.81675	20.48493	19.96523	19.53879	19.89069	19.65021	19.56696	19.63422	2.451219287
nduf-6	22.78166	22.85082	23.23583	22.40324	22.53959	22.80143	21.52287	21.76871	21.62871	22.2156	21.70043	22.73449	2.449163105
C23G10.2	25.04156	25.12078	25.20466	25.38059	25.17196	24.99577	24.78816	24.69215	25.01255	25.04548	24.71746	24.75516	2.442784526
coq-6	22.83794	22.82821	22.98666	23.02948	22.99821	22.8675	22.8369	22.56596	22.80543	22.73604	22.52217	22.56925	2.44046967
ivd-1	26.28472	26.49323	26.17616	26.4181	26.85765	26.58087	27.01656	27.16449	26.89643	26.75341	26.90338	26.74087	2.437430156
Y48A6B.9	22.13959	22.15496	21.64859	21.91647	22.05962	21.80687	22.22168	22.16422	22.28252	22.30303	22.30565	22.41472	2.368310759
mrpl-40	23.1108	23.20947	23.44234	23.84491	24.04785	23.83669	22.81978	23.36492	22.89704	22.92352	22.75223	22.68804	2.354736178
F52C9.3	22.843	22.88988	22.63479	22.55737	22.81673	22.65517	22.85555	22.82551	23.15957	23.08133	23.092	23.16364	2.315914465
mpc-1	25.76293	25.59873	25.3038	25.22046	25.41185	25.15489	25.78079	25.88126	25.75725	25.85962	25.82235	25.59792	2.314699574
dif-1	25.24521	25.29453	24.83397	24.71726	24.95226	24.98758	25.48759	25.29385	25.22892	25.3352	25.43203	25.4144	2.30258303
mrps-16	24.09771	24.0601	24.54638	24.47505	24.40259	24.341	24.00985	23.93096	24.10275	24.14503	23.83852	23.84802	2.254226037
acdh-1	29.22104	29.24079	28.95666	29.02083	28.87488	28.80865	29.24345	29.18556	29.38149	29.2585	29.34535	29.33574	2.198415763
C30F12.2	21.13356	20.80374	21.25554	19.93163	17.32516	20.31502	18.19801	17.88008	17.8499	16.65423	18.54444	18.33993	2.152720061
cisd-3.2	25.92042	26.03693	26.46683	26.41516	26.12605	26.16628	25.62886	25.71293	25.88649	25.96441	25.70963	26.04544	2.098636854
mrpl-55	22.48897	22.44594	23.23714	22.66325	22.6	22.92424	22.10298	22.23174	22.44147	22.5054	21.97496	22.18385	2.084778377
lact-9	20.59962	20.61927	19.91209	19.86023	20.49512	20.29052	20.61708	20.91786	20.81276	20.78852	20.74927	20.65218	1.996560823
ctl-3	20.42322	20.12869	20.32568	20.43544	20.22164	19.85695	19.00308	18.25629	20.109	19.89585	19.63536	18.64713	1.952405411
mrpl-32	22.84015	22.81286	23.56925	23.5648	23.02034	23.10766	22.65325	22.87681	22.63174	22.79447	22.58284	22.66053	1.950632713
pod-2	25.72276	25.62606	24.7919	25.55211	25.64817	25.6239	25.92856	26.11573	26.1109	26.31063	26.11442	25.578	1.902559717
mpst-6	21.93609	21.72289	22.40157	21.64113	23.10736	23.09109	23.08711	23.24989	23.34861	23.26645	22.90864	23.06963	1.886916588
cox-10	21.45357	20.9198	20.74292	20.82999	21.39776	21.22406	21.27914	21.37616	21.6299	21.4725	21.81908	21.69291	1.883641247
crls-1	22.92197	22.8409	21.27194	22.46774	22.96864	22.01657	22.92041	23.01276	23.64857	23.53338	23.23994	23.50061	1.878518159
alh-13	24.71366	24.64235	24.39981	24.47395	24.4743	24.62382	24.94991	24.3373	25.0731	25.03534	25.25636	25.14969	1.803698343
C16C10.1	19.52586	17.90696	16.2833	16.90291	19.15417	17.37687	19.37333	19.75427	19.72203	20.10106	20.13854	18.29725	1.797787734
ant-1.1	30.13063	30.16552	29.44371	29.46313	29.80581	29.69675	29.91112	29.99798	30.72225	30.67697	30.3945	30.25745	1.78800006
tin-10	19.4733	19.56139	20.28926	20.30341	20.17336	20.28389	19.77796	19.68675	19.18556	18.67867	19.14647	19.60343	1.770623387
cox-18	20.7175	20.62566	20.07729	20.18013	20.74884	20.41543	20.7377	20.46421	21.2748	20.86592	21.28714	21.19933	1.755225083
cox-5A	22.51217	22.61371	22.68218	22.60881	22.44444	22.73555	21.72293	21.89002	22.64175	22.24332	22.50695	22.01237	1.733952618
mtx-1	21.51717	21.33693	20.44095	20.19534	20.57732	20.72772	21.17067	21.20786	21.87895	21.88305	21.4914	21.27379	1.675510863
C47E12.2	19.5315	19.77919	17.48552	19.13859	19.61385	17.846	19.65223	19.67041	20.07042	20.17872	20.19234	20.43609	1.643408946
acs-2	22.36165	21.99125	21.60918	22.31086	24.01574	23.5174	23.56818	23.93053	23.55597	23.21442	23.71287	23.64228	1.483480813
Y32H12A.7	21.96778	21.54443	17.95014	19.0764	17.85462	22.1035	21.78019	21.50768	22.22923	22.23409	22.8926	22.0667	1.435083878

sqrd-1	19.86873	18.55417	18.73382	20.38567	18.37298	15.97184	20.97326	18.17332	21.21826	21.06255	20.88519	20.85479	1.423587222

Table S6. Top 60 most significantly altered proteins in the proteomic analysis of WT and Tau-KO mice.

	WT						Tau-KO								
**T: Genes**	**197_1**	**197_2**	**198_1**	**198_2**	**199_1**	**199_2**	**204_1**	**204_2**	**205_1**	**205_2**	**206_1**	**206_2**	**207_1**	**207_2**	**N: -Log Student's T-test p-value WT_TAU_KO**
Gnal	22.2736	22.3883	23.3285	23.327	22.7987	22.6522	17.7293	17.7612	17.1913	17.2987	18.7596	17.7121	16.9434	18.2653	9.19021
Pde1b	25.546	26.0026	25.8319	26.2536	25.7389	26.058	23.2609	23.5257	23.6034	23.7926	23.6168	24.0106	23.8894	23.9065	8.86917
Ptpn5	23.8159	23.629	24.2895	23.8599	24.1504	24.0679	21.3569	21.4733	20.353	20.169	20.6014	20.661	20.7477	20.7767	8.63663
Cpe	23.4697	23.4248	23.3609	23.3315	23.3538	23.3484	24.2298	24.3725	24.3488	24.4887	24.6936	24.7337	24.4646	24.4351	8.40509
Ahcy	25.852	25.8239	25.9004	25.7488	25.8605	25.782	25.4778	25.3482	25.4498	25.369	25.3648	25.4166	25.2896	25.3635	8.10036
Cbr3	24.5431	24.5577	24.6082	24.6213	24.7228	24.7873	23.7541	23.742	23.8464	24.0725	23.9407	23.9333	23.8403	23.8627	7.98867
Cct5	25.3822	25.3832	25.4339	25.3886	25.3968	25.3497	25.1039	25.0094	24.9733	25.0961	25.0978	25.0496	24.9555	25.0325	7.94257
Camk4	24.6251	24.8219	24.7607	24.8727	24.9962	25.0225	23.3386	23.8913	23.8329	23.7582	23.7187	23.5978	23.7767	23.6296	7.74537
Lta4h	25.7125	25.812	25.7807	25.8299	25.5359	25.7329	24.918	25.2028	25.0659	25.0296	25.0235	25.079	24.9102	24.9179	7.61885
Cct8	26.0246	25.9767	26.1414	26.1071	25.9335	25.8411	25.4381	25.3244	25.2657	25.4607	25.2393	25.3461	25.4049	25.3859	7.60073
Prkcb	27.3807	27.5803	27.4739	27.4224	27.2577	27.2247	26.3879	26.4595	26.5348	26.7216	26.605	26.6212	26.6771	26.5832	7.53029
Tiam2	22.3703	22.2397	22.5565	22.3933	22.3286	22.5627	21.3856	21.6477	21.2864	21.4889	21.204	21.2443	21.5708	21.4796	7.50805
Crabp1	21.7929	21.5853	22.7722	22.2178	21.8993	21.6309	18.442	18.1789	17.1461	18.7441	19.3262	17.792	17.4882	17.6973	7.16236
Me2	23.833	23.3701	23.8295	24.0168	24.0812	24.2698	22.2157	22.6272	22.0488	22.2665	22.0653	22.5406	22.3536	22.4032	7.12569
Tanc1	21.2673	21.1423	20.7817	21.0508	21.3275	21.2008	22.0497	22.0598	21.934	22.1771	22.0806	22.1376	21.8749	21.9679	7.02916
Ncl	24.0377	24.2342	24.5146	24.5063	24.2752	24.1879	23.1261	23.197	23.374	23.3474	23.3213	23.4346	23.5019	23.4804	6.95129
Prkcd	21.7908	21.412	21.457	21.1136	21.861	21.6279	19.6851	19.7042	20.0225	20.0339	19.0685	19.4149	19.9145	19.792	6.94238
Crym	27.0084	27.1226	27.4464	27.2705	27.3657	27.2968	28.0771	27.9793	28.1235	28.1963	28.4222	28.3498	28.1456	28.07	6.92601
Actn2	20.5295	20.1589	20.8742	20.6109	21.2457	21.1033	19.154	18.8952	18.8641	19.2689	19.1873	18.8592	18.7496	18.8278	6.90527
Adcy5	20.7692	20.8944	22.4349	22.8451	21.0483	21.6155	18.3736	18.0892	17.6192	18.159	17.9653	17.6638	17.4059	16.985	6.79418
Nt5dc3	22.8357	23.0284	23.0309	22.9608	23.1923	23.0982	23.7786	23.702	24.1039	24.2336	23.9537	23.9123	23.8157	23.8664	6.77178
Elmo2	21.4697	21.3415	21.4953	21.6633	21.534	21.6042	22.2075	22.1933	22.0322	22.0888	22.3757	22.4269	22.3246	22.2809	6.68788
Cpne5	23.9456	24.1388	24.3703	23.7773	24.5947	24.4769	22.9436	22.5233	22.2058	22.5236	22.5017	22.2598	22.6715	22.9253	6.65911
Pfkp	26.5308	26.7293	26.5131	26.503	26.5266	26.5288	26.1686	26.0229	25.963	26.0804	25.8747	26.0267	25.8218	25.9544	6.61055
Vat1l	25.1195	24.9206	25.8736	25.9496	25.342	25.3065	24.0619	23.6157	23.5723	23.3446	23.3052	23.4461	23.7754	23.7606	6.54351

Stum	18.5561	17.2005	17.5829	18.17	18.3391	16.3703	21.2274	21.3345	22.128	22.6829	22.6198	23.1321	21.343	21.4295	6.51322
Dclk1	24.7029	24.8149	24.8102	24.8969	24.9336	24.9599	25.4591	25.3631	25.2344	25.3715	25.2341	25.2823	25.4105	25.3005	6.47834
Chat	21.9138	21.9789	22.4374	22.4171	22.1498	21.8297	20.9611	20.8779	20.1921	20.3271	20.3722	20.1862	20.2835	19.9122	6.46293
Ap2a2	25.1903	25.3553	25.1973	25.1927	25.2501	25.2493	25.5364	25.5207	25.6703	25.7014	25.6903	25.7797	25.7019	25.7045	6.40079
Pgk1	29.1627	29.3506	29.0582	29.2633	29.1434	29.3466	28.5855	28.6887	28.6526	28.7344	28.4732	28.7494	28.5142	28.4777	6.36493
Scamp4	18.5459	18.0354	17.5623	18.2726	20.3383	18.5869	22.1087	21.4323	21.8864	21.9447	22.1051	22.5758	22.127	22.334	6.3365
Iqgap2	23.4031	23.3276	22.9911	23.1429	22.9639	22.852	24.0863	24.0098	24.0363	23.9217	23.9866	24.0751	23.8249	23.803	6.31013
Tppp3	25.5422	25.395	25.5822	25.492	25.9448	25.6875	24.6189	24.3429	24.1519	24.3548	23.8189	23.5655	23.8932	23.6989	6.13505
Lars1	22.6801	22.7454	22.6214	23.0535	22.7906	22.7561	22.2495	22.2571	22.0835	22.1888	22.1634	22.2629	22.2426	22.3087	6.10189
Arhgdib	22.1327	22.4029	22.4925	22.797	22.1927	22.4368	21.2991	21.3962	21.2052	21.4436	21.473	21.7154	21.5895	21.3724	6.04505
Ppp1r7	26.1403	25.9204	25.7883	25.8767	25.8109	25.8972	25.3794	25.2573	25.2107	25.3634	25.3837	25.4301	25.517	25.424	5.95524
Mbnl1	21.9879	22.4978	22.4684	22.5096	22.2519	22.1617	20.915	21.5382	21.2764	21.3304	21.3283	20.9478	21.4033	20.939	5.88979
Rufy3	25.4897	25.5632	25.6122	25.5288	25.3838	25.4311	24.9616	25.0531	24.7878	24.8603	24.5528	24.5486	24.6093	24.6494	5.87321
Rapgefl1	16.5908	16.9403	18.518	17.9924	18.5375	17.573	20.3658	20.064	20.6072	20.7409	20.3015	20.267	19.9155	20.4482	5.81471
Fnbp1	23.0443	23.272	22.9709	23.1825	23.0095	23.0692	22.5714	22.7489	22.5624	22.6082	22.3649	22.5308	22.625	22.5828	5.80715
Srgap3	23.1134	23.0185	22.8919	23.0821	22.9837	23.1404	23.5518	23.688	23.4573	23.5866	23.3102	23.6144	23.4804	23.6081	5.71882
Ppp1r9a	22.3312	22.742	22.7211	23.1201	22.4581	22.8864	21.2123	21.8147	21.673	21.8371	21.3159	21.5605	21.4434	21.328	5.70545
Fam136a	22.1765	22.3655	22.2987	22.2151	21.4848	21.9424	20.7562	20.9899	20.6937	20.5942	21.1369	21.151	20.8128	20.944	5.67561
Th	24.2711	24.3241	24.8153	24.5165	24.8635	24.6242	20.5618	18.9643	18.9186	20.2405	21.1232	20.0713	16.4513	19.765	5.64096
Ddc	23.9918	23.9994	23.8203	24.0921	24.9437	24.8132	22.529	22.2127	22.6819	22.6403	22.8465	22.9245	22.7902	22.8448	5.60852
Adissp	24.7607	24.5167	24.9462	24.7716	24.3623	24.3989	23.9866	23.8455	23.6297	23.4206	23.3916	23.6751	23.6637	23.7079	5.6014
Pitpnc1	22.9623	23.1471	23.0445	23.2213	22.8746	23.183	22.2019	22.2838	21.8997	21.9352	22.3625	22.4969	22.388	22.3384	5.58158
Prkcg	26.1966	26.4088	25.9938	26.0302	26.1344	26.0691	26.6667	26.7024	26.5811	26.7038	26.9059	26.9748	26.8609	26.952	5.49779
Aldh2	25.0291	25.3821	24.7925	24.8367	24.7986	24.8917	24.3355	24.312	23.9254	24.0341	23.9543	24.1506	23.929	23.8443	5.49669
Paics	24.6334	24.5729	24.5808	24.6306	24.3524	24.3453	24.1138	24.0027	24.1567	24.1211	23.8892	23.9575	24.0175	23.9846	5.48446
Mpi	24.262	24.5996	24.2531	24.7081	24.0727	24.3049	23.7453	23.6253	23.6732	23.6035	23.4981	23.6378	23.3937	23.4206	5.47981
Rpl17	24.5977	24.774	25.197	25.5254	24.7909	24.7066	24.0524	23.9501	23.6501	23.56	23.4808	23.6493	23.8156	23.7566	5.47604
Cpne6	25.4117	26.1812	25.3234	25.8621	25.5249	25.8955	26.6001	26.9267	26.644	26.7065	26.9731	27.0778	26.6639	26.8187	5.46325
Nme1	28.7646	29.0428	28.7399	28.7528	28.4982	28.2837	28.1078	27.8308	27.7622	27.8016	27.563	27.6284	27.654	27.5954	5.45388
Pkp2	17.6501	17.6657	17.5142	18.3888	18.4601	19.0168	19.8064	20.3297	19.8727	19.7367	20.1257	20.4715	19.7516	20.2632	5.44392
Plcb1	26.1415	26.2151	26.1591	26.1991	25.8623	26.0318	25.6235	25.7065	25.4837	25.5979	25.5081	25.7169	25.659	25.6977	5.44291
Rps6ka5	21.9119	22.0342	22.5001	22.7092	22.0842	21.9878	20.8028	21.0737	21.2394	21.0601	21.2487	21.2323	21.3398	21.2069	5.41849

Clns1a	19.271	17.4227	17.3649	18.2933	19.4973	19.0581	21.1588	21.2415	20.8455	21.3521	21.2277	21.2376	20.9846	21.074	5.40281
Rapgef2	22.7503	22.5871	22.7493	22.5546	23.0325	22.6568	23.2779	23.2366	23.2891	23.3113	23.495	23.6419	23.5474	23.53	5.39378
Etfa	25.3307	25.6026	25.0151	25.0973	25.347	25.6209	24.5848	24.7152	24.4291	24.5969	24.4954	24.5699	24.2589	24.3593	5.34945

Table S7. Top 60 most significantly altered mitochondrial proteins in the proteomic analysis of WT and Tau-KO mice.

	WT						Tau-KO								
**Genes**	**197_1**	**197_2**	**198_1**	**198_2**	**199_1**	**199_2**	**204_1**	**204_2**	**205_1**	**205_2**	**206_1**	**206_2**	**207_1**	**207_2**	**-log (P-value)**
Me2	23.83304	23.3701	23.82952	24.01682	24.08122	24.26975	22.21567	22.62722	22.04875	22.26655	22.06532	22.54058	22.35356	22.40315	7.1256885
Prkcd	21.79083	21.41203	21.45699	21.11362	21.861	21.6279	19.68514	19.70418	20.02246	20.03393	19.06854	19.41489	19.91449	19.79198	6.9423817
Crym	27.00844	27.1226	27.44644	27.27052	27.36575	27.29675	28.07708	27.97931	28.12345	28.19632	28.42222	28.34978	28.14561	28.06995	6.9260051
Nt5dc3	22.8357	23.02842	23.03093	22.96076	23.19231	23.09823	23.77856	23.70203	24.10386	24.23361	23.95373	23.91228	23.81566	23.86643	6.771776
Chat	21.91376	21.97888	22.43737	22.41712	22.14979	21.8297	20.96109	20.87793	20.19212	20.32715	20.37221	20.18616	20.2835	19.91223	6.462931
Fam136a	22.17654	22.3655	22.29866	22.21515	21.48482	21.94242	20.75619	20.98993	20.6937	20.59422	21.13694	21.15099	20.81284	20.94398	5.675613
Th	24.27108	24.32413	24.81535	24.51654	24.86348	24.62421	20.56176	18.96432	18.91855	20.24052	21.12318	20.07127	16.45127	19.76502	5.6409644
Aldh2	25.02911	25.38212	24.7925	24.83675	24.79858	24.89174	24.33546	24.31203	23.92535	24.03407	23.95426	24.15055	23.92896	23.84431	5.4966948
Nme1	28.76465	29.04277	28.73986	28.75284	28.49821	28.28373	28.10785	27.83079	27.76222	27.80159	27.56295	27.62838	27.65398	27.59545	5.453875
Etfa	25.3307	25.60256	25.01505	25.09734	25.34705	25.62092	24.58477	24.71517	24.42905	24.59687	24.49542	24.56992	24.2589	24.3593	5.3494479
Etfb	25.2539	25.26442	25.09087	24.81053	25.12845	25.12892	24.47745	24.61406	24.41603	24.53202	24.15459	24.38171	24.21356	24.09733	5.330104
Nipsnap2	25.8312	25.99138	25.47059	25.58411	26.07151	26.02761	25.06301	25.2631	24.89026	25.03058	24.63236	24.8587	24.88643	24.90275	5.2519854
Clybl	23.0108	23.02328	22.41881	22.72186	22.52329	22.57587	21.59045	22.08261	22.00025	21.60892	21.46759	21.728	21.61439	21.38595	5.2277889
Ldha	28.07517	28.10951	28.02389	27.77947	28.05152	27.81441	27.63707	27.42075	27.0416	27.26276	27.38216	27.25763	27.24863	27.35241	5.1674428
Acads	22.18827	21.88548	22.03194	21.5817	21.92963	21.95364	21.41285	21.08888	20.49835	21.04931	20.71154	20.57051	20.61661	20.70773	5.0719984
Acadl	25.45402	25.58433	25.13614	25.43463	25.38081	25.47797	24.94869	25.0535	24.7067	24.82109	24.77355	24.5419	24.63464	24.58522	4.9822521
Mtor	22.69385	22.78578	23.10092	22.97177	23.11101	22.99622	23.50899	23.2851	23.5081	23.51847	23.35617	23.49171	23.49304	23.37833	4.94621
Lrpprc	24.18485	24.26136	24.05966	23.90867	24.02291	24.06222	23.58665	23.58891	23.37609	23.49019	23.08668	23.02977	23.03665	23.07554	4.9068714

Dnm3	25.43292	25.30833	25.23363	25.03927	25.37383	25.36662	25.02755	24.72162	24.59102	24.82334	24.62548	24.63032	24.78986	24.81714	4.8439073
Prdx5	27.94922	27.88085	27.71695	27.68502	27.51077	27.36435	26.9948	26.91236	26.75475	26.68173	26.18594	26.63882	26.22012	26.12181	4.8288297
Cpne3	22.67548	22.50151	22.874	22.85451	22.45719	22.27177	21.26662	20.97631	21.71799	21.97561	20.99222	21.56013	21.42366	21.63143	4.8265531
Ass1	25.23028	25.35098	25.27574	25.18742	25.36584	25.2293	24.88264	24.75442	24.84587	24.87141	24.50042	24.55322	24.48546	24.34402	4.783663
Dap3	20.6802	20.42416	21.47035	21.09726	20.63947	20.9373	19.81685	17.80051	18.35347	17.78133	17.31551	18.54744	18.89203	18.938	4.7758294
Sod2	27.50463	27.54158	27.11119	27.38219	27.6987	27.62622	26.76689	26.90097	26.92275	26.91015	26.65811	27.0534	26.62447	26.69465	4.7589373
Suclg1	26.89763	27.23493	26.54426	26.50544	26.94287	27.05408	26.03977	26.18343	25.94538	26.27039	26.15481	26.19083	26.14476	26.20226	4.6239224
Ppm1h	22.99114	23.12644	22.90128	22.72433	23.03939	23.09085	23.38572	23.38927	23.34635	23.38589	23.38602	23.67559	23.47857	23.37907	4.6183272
Mpc1	21.6775	21.23043	23.10089	23.00501	21.65978	21.50743	19.94462	19.38447	19.02751	18.49929	16.8468	18.6126	19.48683	17.74885	4.6177693
Src	18.15933	17.68804	18.54187	18.32631	20.30509	19.5906	20.74936	20.6845	21.20817	21.21251	21.36856	21.62748	21.30325	21.29986	4.5879044
Glod4	25.46852	25.65793	25.59437	25.33546	25.35431	25.24832	24.90906	24.69193	25.14722	25.0873	24.89017	24.92512	24.84858	24.91152	4.4964945
Isoc2a	23.80117	24.06187	23.75971	24.04958	23.80671	23.77014	23.00253	22.83799	22.74067	22.61396	23.10994	22.99233	22.01958	21.93735	4.4513495
Ogt	24.0623	24.25077	23.96097	23.94983	24.309	24.26833	24.4621	24.55835	24.49969	24.54464	24.78526	24.77704	24.68668	24.59961	4.4269618
Hmgcl	22.69278	22.578	22.57195	22.32671	22.24679	22.73607	21.87831	22.14129	21.67266	21.90397	21.6165	21.78371	21.35864	21.42087	4.4073157
Bag5	22.54536	22.36434	22.97206	22.85666	22.56176	22.64467	21.84165	21.81307	21.85409	21.94366	22.24386	22.12654	22.06066	22.21335	4.2995341
Acaca	20.00347	19.93166	20.07791	19.99354	20.11338	19.92246	20.64001	20.1717	20.58214	20.7208	20.33673	20.44394	20.49788	20.68996	4.2933418
Ndufb10	19.97962	20.01212	21.70496	22.01769	20.32389	20.06897	16.57674	18.71754	16.45022	17.40407	17.78109	19.02195	17.38298	18.1466	4.2075984
Gclc	23.58314	23.85159	23.54716	23.68335	23.42825	23.52815	23.30343	23.39592	22.96492	23.00977	23.11804	23.07327	23.07469	23.01046	4.1951873
Stxbp1	28.22311	28.55822	28.38619	28.53462	28.28472	28.37821	27.95982	28.0672	27.99294	28.13648	27.71383	27.76336	27.69317	27.74294	4.1722129
Usp15	23.24994	23.16954	23.082	23.26262	23.03394	22.95068	23.01177	22.62698	22.46482	22.52322	22.47326	22.53396	22.60955	22.70331	4.1600841
Hars1	25.4745	25.72692	25.67459	25.4982	25.24713	25.3361	25.23177	25.01241	24.73793	25.0054	24.96445	24.98919	24.97258	25.00906	4.148371

Park7	27.0592	27.22527	27.23078	27.26808	27.3471	27.29696	26.95955	26.97828	26.87164	26.80051	26.67873	26.7089	26.60803	26.46305	4.1469804
Ckb	29.80309	30.21319	29.7966	29.79473	30.46931	30.32594	29.41198	29.31308	29.40796	29.48621	29.43915	29.52128	29.40153	29.3547	4.1445061
Echs1	25.29392	25.43793	24.96584	25.47367	25.13888	25.4612	24.92131	24.90959	24.74021	24.77401	24.59375	24.85149	24.6703	24.54399	4.1334868
Mdh1	30.65911	30.96228	30.42551	31.05559	30.57539	30.92054	29.9431	30.42933	29.90337	29.96501	30.0555	30.24245	29.98749	30.21536	4.0982926
Nt5c3a	21.40664	21.6579	22.03485	21.65353	21.88815	21.51929	20.14607	20.56885	20.60383	20.73168	18.72918	19.89714	19.80242	20.16772	4.0874792
Rtn4ip1	21.86621	21.3236	21.43542	21.28804	21.22577	20.84288	20.78616	19.68845	17.40029	17.86017	17.55463	17.83028	18.09943	17.5186	4.044586
Fth1	23.96077	24.01752	24.48091	23.94204	23.37119	23.227	22.4277	22.54706	22.20071	22.47991	23.09893	23.12314	22.1136	22.3931	4.0438706
Cryab	23.72686	24.01078	24.08793	23.78341	24.54887	24.12662	23.01463	22.71139	23.39968	23.64533	23.06017	23.11477	23.24011	22.88608	4.0409142
Etnppl	21.19875	21.11212	20.82841	20.85526	22.0006	22.04461	20.36625	20.22065	19.86989	20.18825	20.17738	20.18622	19.89343	20.36352	4.0287295
Qdpr	27.67308	27.74374	27.69843	27.55451	27.96224	27.99759	27.29243	27.42696	27.31213	27.45933	27.04892	27.11536	27.06966	26.94258	3.9975406
Hebp1	23.69616	23.74185	23.73934	23.59446	24.22692	24.35658	23.11977	23.03646	23.39045	23.33884	23.0809	23.28495	23.13686	23.21622	3.9759361
Eci1	24.26605	24.17366	24.07848	24.01226	23.93559	24.01959	23.48687	23.35026	23.26554	23.36558	22.59397	22.83165	22.26287	22.42243	3.9366118
Plaat3	20.37673	18.58287	20.55539	19.69426	21.01511	20.0755	17.32846	18.66961	18.04611	17.74615	16.66122	18.49243	17.92301	18.08347	3.8720043
Opa1	22.53202	22.16755	23.02157	22.73529	22.11804	22.02572	21.81602	21.52569	21.05867	21.08944	21.41784	21.22792	20.43748	20.588	3.845496
Mat2b	24.04066	24.31726	23.88054	24.08432	23.61189	23.84437	23.37764	23.69573	23.18907	23.32516	23.32504	23.41957	23.41338	23.45548	3.8368632
Gpx1	24.00437	23.96876	24.04702	23.6163	23.87967	23.62637	23.60118	23.20082	23.15908	22.73736	22.76736	22.43453	22.50208	22.54783	3.8117294
Mecr	23.36366	23.11744	22.93747	22.57611	23.18178	23.15729	22.68178	22.65142	22.28183	22.31827	22.11322	22.16574	21.98253	22.04734	3.7846123
Fh	26.83516	26.78325	26.49769	26.33307	26.96834	26.85956	26.26792	26.15387	26.34407	26.33274	26.11275	26.16976	26.03662	26.04748	3.7827907
Lap3	25.14339	25.2453	25.0532	24.94416	25.12197	25.15486	24.73901	24.42815	24.78479	24.97865	24.47104	24.71783	24.64585	24.6718	3.7683073
Ndufs1	21.72916	21.36274	22.85193	23.00343	22.58651	22.35303	21.15819	20.672	21.07611	21.16118	21.14906	21.18808	20.41601	20.69947	3.7628902
Letm1	19.24819	19.60589	21.03396	20.36217	20.8793	20.48019	19.09449	18.87184	17.06175	17.20618	18.66239	17.12375	18.25518	17.91142	3.7624568

SI References

1. H. N. Dawson *et al.*, Inhibition of neuronal maturation in primary hippocampal neurons from tau deficient mice. *J Cell Sci* **114**, 1179-1187 (2001).
2. A. Ahier *et al.*, Affinity purification of cell-specific mitochondria from whole animals resolves patterns of genetic mosaicism. *Nat Cell Biol* **20**, 352-360 (2018).
3. I. L. Ferreir a, C. Carmo, L. Naia, S. I. Mota, A. Cristina Rego, "Assessing Mitochondrial Function in In Vitro and Ex Vivo Models of Huntington's Disease" in Huntington's Disease, S. V. Precious, A. E. Rosser, S. B. Dunnett, Eds. (Springer New York, New York, NY, 2018), 10.1007/978-1-4939-7825-0_19, pp. 415-442.
4. D. C. David *et al.*, Proteomic and functional analyses reveal a mitochondrial dysfunction in P301L tau transgenic mice. *J Biol Chem* **280**, 23802-23814 (2005).
5. N. Otsu, A Threshold Selection Method from Gray-Level Histograms. . *2EEE TRANSACTIONS ON SYSTREMS, MAN, AND CYBERNETICS,* **smc-9** (1979).
6. W.-H. Tsai, Moment-preserving thresolding: A new approach. *Computer Vision, Graphics, and Image Processing* **Volume 29**, 377-393 (1985).
7. V. Demichev, C. B. Messner, S. I. Vernardis, K. S. Lilley, M. Ralser, DIA-NN: neural networks and interference correction enable deep proteome coverage in high throughput. *Nat Methods* **17**, 41-44 (2020).
8. S. Tyanova *et al.*, The Perseus computational platform for comprehensive analysis of (prote)omics data. *Nat Methods* **13**, 731-740 (2016).
9. M. Samiotaki and K. Palikaras, Differential Proteomics of Ptl-1 KO and Control C.elegans. PRIDE. https://www.ebi.ac.uk/pride/archive/projects/PXD073360. 21 January 2026.
10. Martina Samiotaki and Ioannis Sotiropoulos, Comparative Proteomic Profiling of Tau-Knockout and Wild-Type Mouse Hippocampus. PRIDE. https://www.ebi.ac.uk/pride/archive/projects/PXD073466. 23 January 2026.
